# Disentangling the molecular mystery of tumour–microbiota interactions: Microbial metabolites

**DOI:** 10.1002/ctm2.70093

**Published:** 2024-11-20

**Authors:** Yu‐Fei Duan, Jia‐Hao Dai, Ying‐Qi Lu, Han Qiao, Na Liu

**Affiliations:** ^1^ State Key Laboratory of Oncology in South China, Guangdong Key Laboratory of Nasopharyngeal Carcinoma Diagnosis and Therapy, Guangdong Provincial Clinical Research Center for Cancer Sun Yat‐sen University Cancer Center Guangzhou PR China

**Keywords:** cancer, functional mechanisms, microbial metabolites, microbial metabolomics

## Abstract

The profound impact of the microbiota on the initiation and progression of cancer has been a focus of attention. In recent years, many studies have shown that microbial metabolites serve as key hubs that connect the microbiome and cancer progression, but the underlying molecular mechanisms have not been fully elucidated. Multiple mechanisms that influence tumour development and therapy resistance, including disrupting cellular signalling pathways, triggering oxidative stress, inducing metabolic reprogramming and reshaping tumour immune microenvironment, are reviewed. Focusing on recent advancements in this field, this review also summarises the methodological framework of studies regarding microbial metabolites. In this review, we outline the current state of research on tumour‐associated microbial metabolites and describe the challenges in future scientific research and clinical applications.

**Key points:**

- Metabolites derived from both gut and intratumoural microbiota play important roles in cancer initiation and progression.
- The dual roles of microbial metabolites pose an obstacle for clinical translations.
- Absolute quantification and tracing techniques of microbial metabolites are essential for addressing the gaps in studies on microbial metabolites.
- Integrating microbial metabolomics with multi‐omics transcends current research paradigms.

## Graphical abstract

Duan et al. comprehensively summarise the functional mechanisms of microbial metabolites in tumour development and their clinical translational potential as promising biomarkers and therapeutic targets. The methodological framework of microbial metabolomics research is thoroughly discussed. This review also highlights pressing issues in microbial metabolites research that require resolution and provides new insights for future scientific investigation.

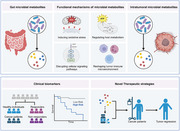

## 1 INTRODUCTION

The human microbiome constitutes a complex multi‐kingdom community that influences the development and progression of multiple types of cancer.^1^ With advancements in metabolomics based on mass spectrometry (MS) and nuclear magnetic resonance (NMR) technologies, microbial metabolites have been identified as key effector molecules that link the microbiome to cancer and have garnered widespread attention.^2^ For instance, short‐chain fatty acids (SCFAs), especially butyrate, are representative microbial metabolites that enhance therapeutic effects; supplementation with butyrate‐producing *Clostridium butyricum* 588 (CBM588) can improve clinical outcomes in trials.^3^ In addition to their potent anticancer effects, some microbiota‐derived metabolites also exhibit carcinogenic properties. Various metabolites produced by tumour‐promoting bacteria have been confirmed as potent regulatory molecules in tumour malignant progression, such as succinic acid derived from *Fusobacterium nucleatum* (*F. nucleatum*) and indolimines derived from *Morganella morganii*.^4^, ^5^ Despite remarkable progress, the specific biological effects and molecular mechanisms of microbial metabolites on tumours remain elusive.

In this review, we summarise the metabolic processes of major tumour‐associated microbial metabolites and their dual roles in tumour development and therapy, along with discussions of their potential prospects as biomarkers and targets for novel therapeutic strategies. Furthermore, we highlight methodological framework and pressing issues in microbial metabolite research, emphasising potential goals of future scientific investigations.

## 2 FUNCTIONAL MECHANISMS OF THE PRIMARY TUMOUR‐ASSOCIATED MICROBIAL METABOLITES

**FIGURE 1:**
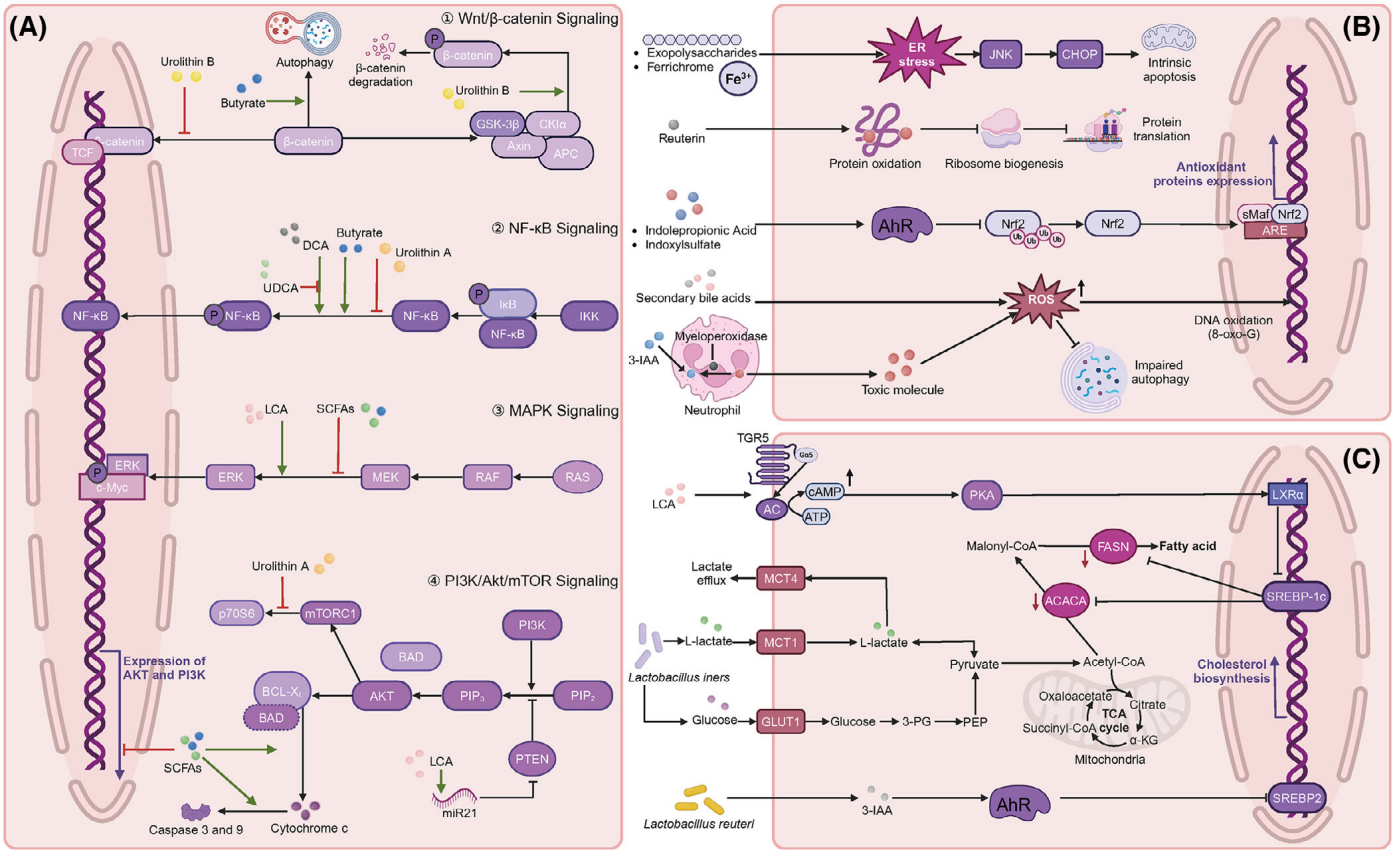
Microbial metabolites influence tumour progression via signalling disruption, oxidative stress and metabolic regulation. (A) Disrupting cellular signalling pathways. Microbial metabolites serve as pivotal mediators linking tumour development and signalling pathways, such as the Wnt/β‐catenin pathway, the NF‐κB pathway, the MAPK pathway and the PI3K/Akt/mTOR pathway. (B) Triggering oxidative stress. Microbial metabolites instigate oxidative stress, subsequently triggering mitochondrial autophagy, DNA damage and protein oxidation. (C) Inducing metabolic reprogramming. Microbial metabolites fundamentally alter host metabolic pathways, including cholesterol biosynthesis, lipid synthesis and lactate metabolism. AC, adenylate cyclase; ACACA, acetyl‐CoA carboxylase alpha; AhR, aryl hydrocarbon receptor; Akt, serine/threonine kinase; APC, adenomatous polyposis coli; ARE, antioxidant response element; BAD, Bcl2‐associated agonist of cell death; BCL‐XL, B‐cell lymphoma‐extra large; cAMP, cyclic adenosine monophosphate; CHOP, CCAAT/enhancer‐binding protein homologous protein; CKIα, casein kinase Iα; DCA, deoxycholic acid; 8‐oxoG, 8‐oxoguanine; ERK, extracellular regulated kinase; ER stress, endoplasmic reticulum stress; FASN, fatty acid synthase; GLUT1, glucose transporter type 1; GSK3β, glycogen synthase kinase 3β; IκB, inhibitor of NF‐κB; IKK, IκB kinase; JNK, Jun N‐terminal kinase; LCA, lithocholic acid; LXRα, liver X receptorα; MAPK, mitogen‐activated protein kinase; MCT, monocarboxylate transporter; MEK, MAPK/ERK kinase; mTOR, mammalian target of rapamycin; mTORC1, mechanistic target of rapamycin complex 1; NF‐κB, nuclear factor kappa B; Nrf2, nuclear factor erythroid 2‐related factor 2; PEP, phosphoenolpyruvate; PIP2, phosphatidylinositol bisphosphate; PI3K, phosphatidylinositol‐3‐kinase; PKA, protein kinase A; p70S6K, 70 kDa ribosomal protein S6 kinase; PTEN, phosphatase and tensin homolog; RAF, rapidly accelerated fibrosarcoma; RAS, rat sarcoma; ROS, reactive oxygen species; SCFAs, short‐chain fatty acids; sMaf, small musculoaponeurotic fibrosarcoma oncogene homolog protein; SREBP, sterol regulatory element binding protein; TCA cycle, tricarboxylic acid cycle; TCF, T‐cell factor; TGR5, G‐protein‐coupled bile acid receptor 1; 3‐IAA, indole‐3‐acetic acid; 3‐PG, 3‐phosphoglycerate; UDCA, ursodeoxycholic acid; Wnt, wingless‐related integration site.

**FIGURE 2:**
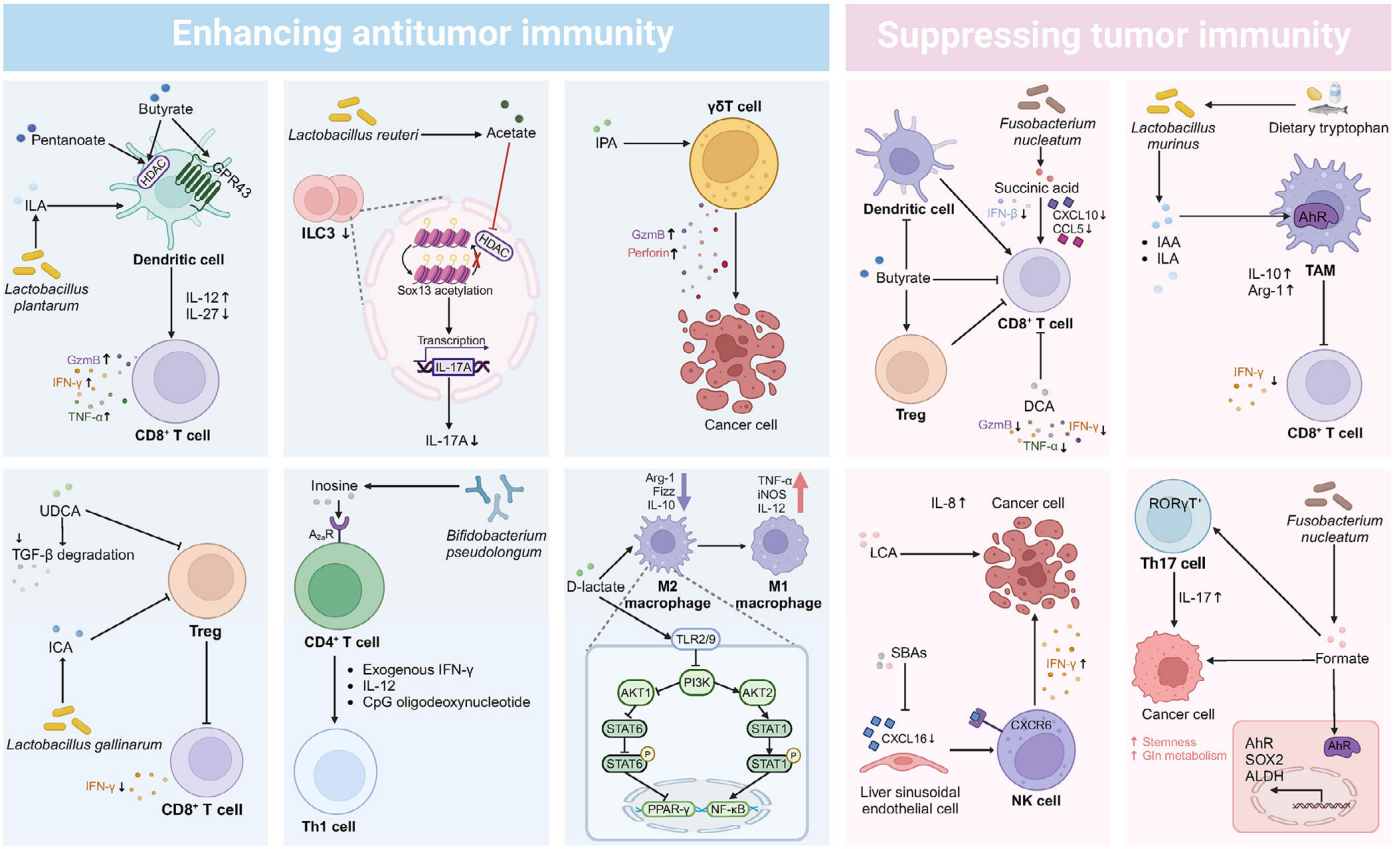
Dual roles of microbial metabolites in reshaping the tumour immune microenvironment. Microbial metabolites possess a remarkable capacity for immune modulation through modulating the differentiation programs of immune cells and the secretion patterns of effector molecules, enabling them to dually reshape the tumour immune microenvironment towards either anti‐tumour or pro‐tumour properties. AhR, aryl hydrocarbon receptor; Akt, serine/threonine kinase; ALDH, acetaldehyde dehydrogenase; Arg‐1, arginase‐1; A2AR, adenosine 2A receptor; CCL, C‐C motif ligand; CXCL, C‐X‐C motif chemokine ligand; CXCR, C‐X‐C motif chemokine receptor; DCA, deoxycholic acid; Fizz, found in inflammatory zone 1; GPR, G protein‐coupled receptor; Gzm B, granzyme B; HDACs, histone deacetylases; IAA, indoleacetic acid; ICA, indole‐3‐carboxylic acid; IFN‐γ, interferon‐γ; IL, interleukin; ILA, indole‐3‐lactic acid; ILC3s, type 3 innate lymphoid cells; iNOS, inducible nitric oxide synthase; IPA, 3‐indolepropionic acid; LA, indole‐3‐lactic acid; LCA, lithocholic acid; NF‐κB, nuclear factor kappa B; NK cell, natural killer cell; PI3K, phosphatidylinositol‐3‐kinase; PPAR, peroxisome proliferator‐activated receptors; RORγΤ, retinoic acid receptor‐related orphan receptor γΤ; SBAs, secondary bile acids; Sox, SRY (sex‐determining region Y)‐box transcription factor; STAT, signal transducers and activators of transcription; TAM, tumour‐associated macrophage; TGF‐β, transforming growth factor β; Th, helper T cell; TLR, Toll‐like receptor; TNF‐α, tumour necrosis factor‐α; Treg, regulatory T; UDCA, ursodeoxycholic acid.

Except for local impacts on intestinal cancers, numerous studies have demonstrated the long‐distance effects of gut microbial metabolites on cancers that are outside the gastrointestinal tract. In addition, intratumoural microbial metabolites have gradually garnered significant attention due to their more local and direct effect on tumours. With a deeper understanding of host–microbe metabolite interactions, the anti‐tumour (Table 1) and pro‐tumour (Table 2) mechanisms of various microbial metabolites have been elucidated, including disrupting cellular signalling pathways (Figure 1A), triggering oxidative stress (Figure 1B), inducing metabolic reprogramming (Figure 1C) and reshaping the tumour immune microenvironment (Figure 2).

**TABLE 1:** The main mechanisms and circumstances of microbial metabolites in anti‐tumour effect.

Metabolites	Structural formula	Derive from	Mechanisms	Cancer type	Conditions	Level of evidence	Refs.
SCFAs	/	Gut microbiota	Downregulates the expression of PI3K/Akt, promotes the release of cytochrome c, and subsequently activates caspase‐9 and caspase‐3	Colorectal cancer (CRC)	/	Cell lines (HCT‐116), mouse models	^6^
		Gut microbiota	Binds to GPR43 on DCs and prevents them from producing IL‐27 which can lead to CD8^+^ T cells exhaustion	CRC	/	Mouse models (ApcMin^−/+^Ffar2^−/−^ mice)	^7^
		*Gut Megasphaera massiliensis*‐derived pentanoate and gut microbiota‐derived butyrate	Enhances CD8^+^ T cell anti‐tumour activity by targeting HDACs, resulting in metabolic and epigenetic reprogramming that increase effector molecules like CD25, IFN‐γ and TNF‐α	Melanoma and pancreatic cancer	/	Mouse models	^8^
Butyrate	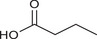	Gut microbes (*Faecalibaculum rodentium* and *Holdemanella biformis* in human)	Inhibits the calcineurin‐mediated activation of the NFATc3 transcription factor	CRC	Millimolar concentrations of butyrate	Cell lines (HT‐29 and Caco‐2), mouse models	^9^
		/	Reduces the secretion of VEGF and tumour progression by inhibiting the activity on HDACs	Glioblastoma	/	Cell lines (T98G, U251MG and U87MG)	^10^
		/	Enhances TLR4‐mediated NF‐κB signalling activation, thereby boosting the anti‐tumour innate immunity	CRC	/	Cell lines (SW480 and CT26)	^11^
		/	Regulates the ratio of BAX/BCL‐2 to induce apoptosis and lower the expression of β‐catenin, P53, and P21	CRC	Millimolar concentrations of butyrate	Cell lines (WiDr, C2BBe1 and LS1034), mouse models	^12^
		/	Promotes the autophagic degradation of β‐catenin weakening the Wnt signalling	CRC	/	Cell lines (HCT116 and SW620)	^13^
		/	Inhibits xCT‐dependent glutathione synthesis through class I HDAC inhibition, thus boosting the ferroptosis	CRC	Millimolar concentrations of butyrate	Cell lines (HCT116, SW480, SW620 and RKO), mouse models, organoid	^14^
		Gut microbiota	Increases H3K27 acetylation at the promoter region of Pdcd1 and Cd28 in CTLs, thereby promoting the expression of PD‐1/CD28	Non–small‐cell lung cancer	/	Cell lines (Jurkat and B16‐F0), mouse models, human blood samples	^15^
		Gut microbes (*Roseburia intestinalis*)	Activates CTLs through directly binding to TLR5 and activating NF‐κB signal	CRC	/	Cell lines (HCT116, LoVo and SW480), mouse models, organoid, human faecal samples	^16^
		Gut microbiota	Suppresses the expression of PD‐L1 and IL‐10 in TAMs	Gastric cancer	/	Cell lines (AGS), mouse models, human blood and cancer tissue samples	^17^
		Gut microbiota	Facilitates CD8^+^ T cell responses through IL‐12 signalling depends on ID2	CRC	Low‐dose butyrate, high‐dose propionate	Cell lines (MC38 and EG7), mouse models, human blood sample	^18^
		Intratumoural *Fusobacterium nucleatum*	Inhibits HDAC3/8 activity in CD8^+^ T cells, leading to increased H3K27 acetylation at the TBX21 promoter, which suppresses elevated PD‐1 expression	Microsatellite stable CRC	/	Cell lines (HT29 and HCT116), mouse models, human faecal and cancer tissue sample	^19^
Acetate	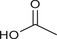	Gut microbiota, particularly *Lactobacillus reuteri*	Reduces IL‐17A production in hepatic ILC3s by inhibiting HDACs and increasing Sox13 acetylation at site K30	Hepatocellular carcinoma	/	Mouse model, human serum sample	^20^
		Gut microbes (*Blautia*)	Restore the function of impaired CD8^+^ T cells	Breast cancer	Co‐existing depression	Cell lines (4T1 and E077), mouse models, human faecal, blood and breast tissue samples	^21^
		/	Is taken up by CD8^+^ T cells and NK cells and converted into acetyl‐CoA by ACSS2, thereby promoting immune cells proliferation and function	Breast cancer	Under glucose deficiency	Cell lines (4T1, A7C11, T11, T12 and Brpkp 110), mouse models	^22^
Propionate	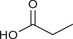	Gut microbiota	Coordinates proteasomal degradation via HECTD2 upregulation to target EHMT2	CRC	/	Cell lines (HCT116 and LS174T), mouse models	^23^
		/	Inhibit the MAPK signalling pathway and Hippo‐Yap via GPR41 and GPR43, respectively	Breast cancer	/	Cell lines (MDA‐MB‐231 and MCF‐7)	^24^
IAA	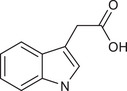	Gut *Lactobacillus reuteri*	Activates AhR to inhibit SREBP2 post‐translationally and reverse the tumourigenesis	Liver cancer	/	Mouse models, human faecal samples	^25^
		Gut *Bacteroides fragilis* and *Bacteroides thetaiotaomicron*	Is converted into a kind of toxic molecule by myeloperoxidase in neutrophils, leading to the accumulation of ROS and downregulation of autophagy	Pancreatic cancer	/	Cell lines (KPC), mouse models, human serum and faecal samples	^26^
I3A	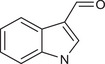	Intratumoural *Lactobacillus reuteri*	Activates AhR to promote IFN‐γ‐producing CD8^+^ T cells	Melanoma	/	Cell lines (B16‐F0, YUMM1.7, MC38 and HT29), mouse models, human serum samples	^27^
ICA	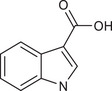	*Gut Lactobacillus gallinarum*	Competitively inhibits Kyn‐induced AhR activation, thereby suppressing CD4^+^ Treg differentiation and enhancing CD8^+^ T cell function	CRC	/	Cell lines (MC38 and CT26), mouse models	^28^
IPA	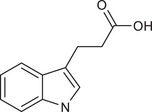	Gut microbiota	Enhances the cytotoxic capacity of γδ T cells and stimulates the release of granzyme B and perforin	Hepatocellular carcinoma	/	Cell lines (HepG‐2), mouse models	^29^
		Gut *Lactobacillus johnsonii* collaborates with *Clostridium sporogenes*	Modulates the stemness program of CD8^+^ T cells and facilitates the generation of progenitor exhausted CD8^+^ T cells by increasing H3K27 acetylation at the super‐enhancer region of Tcf7	In pan‐cancer, including melanoma, breast cancer and CRC	/	Cell lines (MC38, B16‐F10 and 4T1), mouse models, organoid, human tissues and faecal samples	^30^
ILA	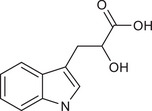	Gut *Lactobacillus reuteri*	Targets the RORγt and downregulates the IL‐17 signalling pathway to inhibit Th17 cell differentiation	CRC	/	Cell lines (HCT116 and LS174T), mouse models	^31^
		Gut *Lactobacillus plantarum*	Accelerates IL12a production in DCs and transcriptionally inhibits SAA3 expression related to cholesterol metabolism of CD8^+^ T cells	CRC	/	Cell lines (MC‐38), mouse models	^32^
LCA	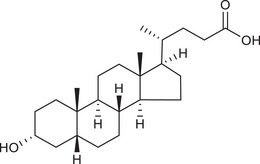	Gut microbiota	Reduces Bcl‐2 expression and Akt phosphorylation while increasing TGR5 and p53 expression	Breast cancer	/	Cell lines (MCF‐7 and MDA‐MB‐231)	^33^
DCA	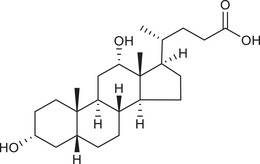	Gut microbiota	Upregulates MUC2 mRNA expression and decreases the expression of Snail and MMP9	Gastric carcinomas	At a higher concentration (200 µM)	Cell lines (SNU‐216 and MKN45), resected human GC samples	^34^
		Gut microbiota	Decreases miR‐92b‐3p expression in an m6A‐dependent post‐transcriptional modification manner, which increased the protein level of the phosphatase and tensin homolog, and subsequently inactivated the PI3K/AKT signalling pathway	Gallbladder cancer	At concentrations of µM	Cell lines (NOZ, GBC‐SD and EGH1), mouse models, resected human GBC samples	^35^
UDCA	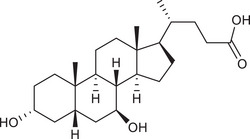	Gut microbiota	Inhibits NF‐κB signalling pathway by suppressing the function of IKK and blocking the translocation of NF‐κB to the nucleus	CRC	/	Cell lines (HCT116)	^36^
		Gut microbiota	Suppresses the upregulation of Cox‐2 by decreasing its transcriptional regulator C/EBP β	CRC	/	Cell lines (HCA‐7), mouse models	^37^
		Gut microbiota	Induces CHIP‐mediated TGF‐β degradation to restrain Treg cells differentiation	Melanoma, CRC and lung cancer	/	Cell lines (B16‐F10, MC38 and LLC), mouse models, human blood and lung tissue samples	^38^
TMAO	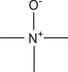	Gut microbiota	Enhances the type I IFN pathway, induces tumour‐associated macrophage phenotypes and activates effector T cells	Pancreatic cancer	/	Cell lines (mouse PDAC cell clones), mouse models	^39^
		Gut and intratumoural *Clostridiales*	Activates the PERK to induce GSDME‐mediated pyroptosis of tumour cells and enhances CD8^+^ T cell‐mediated immunity	Triple‐negative breast cancer	High TMAO levels in plasma and tumour	Cell lines (4T1, 66cl4, HEK293T and MDA‐MB‐23), mouse models, human tumour and blood samples	^40^
Inosine	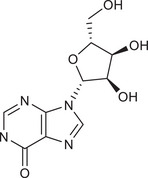	Gut *Bifidobacterium pseudolongum*	Promotes Th1 differentiation through an inosine‐A2AR‐cAMP‐PKA signalling pathway	CRC, bladder cancer and melanoma	In the presence of exogenous IFN‐γ and co‐stimulation, for example, CpG oligodeoxynucleotide	Cell lines (MC38, B16‐F10, MB49), mouse models	^41^
Lactic acid	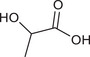	Gut microbiota (D‐lactate)	Inhibition of PI3K/ Akt pathway and activation of NF‐kB pathway to transform M2 tumour‐associated macrophages into M1	HCC	/	Cell lines (Hepa1‐6), mouse models, ex vivo studies	^42^
		Vaginal *Lactobacillus* spp., particularly *Lactobacillus iners*	Activates the Wnt pathway through the lactate‐Gpr81 complex, which increases the level of core fucosylation in epidermal cells	Cervical cancer	/	Cell line (SiHa, HeLa and 293T), human vaginal secretions samples	^43^
Urolithin A	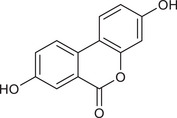	Gut microbiota	Decreases the phosphorylation of AKT and p70S6K	Pancreatic cancer	/	Cell lines (MiaPaCa2, PANC1, AsPC1, CFPAC1, Capan1, Capan2, SW1990, HPAC and BxPC3), mouse models	^44^
		Gut microbiota	Induces senescence‐associated β‐galactosidase activity, inducing p53‐dependent cellular senescence	CRC	/	Cell lines (HCT‐116, Caco‐2, HT‐29 and CCD18‐Co)	^45^
		Gut microbiota	Induces Pink1‐dependent mitophagy in CD8^+^ T cells by enhancing Wnt signalling and promotes the formation of T memory stem cells	CRC	/	Mouse models, tumour sample	^46^

Abbreviations: ACSS2, acyl‐CoA synthetase short‐chain family member 2; AhR, aryl hydrocarbon receptor; Akt, serine/threonine kinase; A2AR, adenosine 2A receptor; BAX, BCL2‐associated X; BCL‐2, B‐cell lymphoma‐2; cAMP, cyclic adenosine monophosphate; C/EBPβ, CCAAT/enhancer binding protein β; CHIP, carboxyl terminus of Hsc70‐interacting protein; Cox‐2, cyclooxygenase‐2; CTL, cytotoxic CD8^+^ T cells; DC, dendritic cell; DCA, deoxycholic acid; EHMT2, euchromatic histone‐lysine N‐methyltransferase 2; GPR, G protein‐coupled receptor; GSDME, gasdermin E; HDACs, histone deacetylases; HECTD2, HECT domain E3 ubiquitin protein ligase 2; H3K27, histone H3 lysine 27; IAA, indole acetic acid; ICA, indole‐3‐carboxylic acid; ID2, inhibitor of DNA binding 2 gene; IFN‐γ, interferon‐γ; IKK, IκB kinase; IL, interleukin; ILA, indole‐3‐lactic acid; ILC3s, type 3 innate lymphoid cells; IPA, 3‐indolepropionic acid; I3A, indole‐3‐aldehyde; LCA, lithocholic acid; MAPK, mitogen‐activated protein kinase; MMP9, matrix metalloproteinase 9; MUC2, mucin 2; NFATc3, nuclear factor of activated T cells 3; NF‐κB, nuclear factor kappa B; PERK, endoplasmic reticulum stress kinase; Pink1, PTEN‐induced putative kinase 1; PI3K, phosphatidylinositol‐3‐kinase; PKA, protein kinase A; RORγt, RAR‐related orphan receptor γt; ROS, reactive oxygen species; SAA3, serum amyloid A3; SCFAs, short‐chain fatty acids; Sox13, SRY (sex‐determining region Y)‐box 13; SREBP2, sterol regulatory element binding protein 2; TAMs, tumour‐associated macrophages; TBX21, T‐box transcription factor 21; TGF‐β, transforming growth factor β; TGR5, G‐protein‐coupled bile acid receptor 1; Th, helper T cell; TLR, Toll‐like receptor; TMAO, trimethylamine‐N‐oxide; TNF‐α, tumour necrosis factor‐α; Treg, regulatory T; UDCA, ursodeoxycholic acid; VEGF, vascular endothelial growth factor; Wnt, wingless‐related integration site.

**TABLE 2:** The main mechanisms and circumstances of microbial metabolites in pro‐tumour effect.

Metabolites	Structural formula	Derive from	Mechanisms	Cancer type	Conditions	Level of evidence	Refs.
SCFAs	/	Gut microbiota	Stimulates the production of IGF1 to promote prostate cancer via activating the MAPK and PI3K signalling	Prostate cancer	/	Cell lines (DU145 and 22Rv1), mouse models	^47^
Butyrate	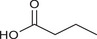	Gut microbiota	Induces aberrant proliferation and transformation of colon epithelial cells by regulating the activity of β‐catenin	Colorectal cancer (CRC)	/	Mouse models (Apc^Min/+^ and MSH2^−/−^ mice)	^48^
		Gut microbiota	Elicit senescence‐like phenotypes	CRC	/	Mouse models (Apc^Δ14/+^ mice), human faecal sample	^49^
		Intratumoural *Roseburia*	Increases expression of H19 in tumour cells through its inhibitory effect on HDAC2, increasing H3K27 acetylation and inducing M2 macrophage polarisation	Lung cancer	/	Cell lines (A549, NCI‐H1299, LLC and RAW 264.7), mouse models, organoid, human lung cancer tumour and plasma samples	^50^
		/	Undermines the radiotherapy‐induced antigen presentation to DCs, thereby impairing the tumour‐killing effect mediated by CD8^+^ T cells	Melanoma, lung cancer and cervical cancer	/	Cell lines (B16 and TC‐1), mouse models	^51^
		Gut microbiota	Restrains upregulation of CD80/CD86 on DCs and ICOS on T cells, accumulation of tumour‐specific T cells and memory T cells	Metastatic melanoma	High concentrations of butyrate	Cell lines (MC38, CT26 and MCA101_OVA_), mouse models, ex vivo studies, human blood, serum and faecal samples	^52^
		Gut microbiota	Elicits an immunosuppressive response by increasing Tregs number and impairing the function of CD8^+^ T cells	NAFLD‐HCC	/	Human faecal and serum samples	^53^
Acetate	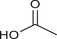	Gut microbiota	Upregulates glutamine and UDP‐GlcNAc levels and enhances protein O‐GlcNAcylation, while hyper‐O‐GlcNAcylation of eukaryotic elongation factor 1A1 promotes cell proliferation	Hepatocellular carcinoma	Fructose supplementation	Cell lines (MHCC‐97H and HEK293), mouse models, human hepatocarcinoma cancer samples	^54^
		/	Induces a hyperacetylated state of histone H3 in hypoxic cells to activate lipogenic genes ACACA and FASN expression, while also functions as an epigenetic metabolite	Hepatocellular carcinoma	Under hypoxic stress	Cell lines (HepG2), human hepatocarcinoma cancer samples	^55^
		/	Is converted into acetyl‐CoA through ACSS2, resulting in an increased acetylation level of lysine 148 on c‐Myc, which promotes the stability of the c‐Myc and subsequently activates the transcription of PD‐L1, LDHA, MCT1 and cyclin D1	Non–small‐cell lung cancer	/	Cell lines (A549 and H1299), mouse models, tumour tissue samples	^56^
Propionate	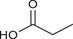	/	The accumulation of metabolic by‐products methylmalonic acid promotes cancer cell invasiveness	Breast cancer and lung cancer	High concentrations of propionate	Cell lines (MCF‐10A, HCC1806 and MDA‐MB‐231), mouse models	^57^
IAA	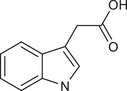	Gut *Lactobacillus*	Activates the AhR in TAMs promoting the expression of Arg1 and IL‐10, and inhibits IFN‐γ expression in CD8^+^ T cells	Pancreatic ductal carcinoma	/	Human monocyte‐derived macrophages, mouse models	^58^
IDA	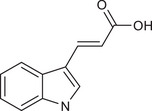	Gut *Peptostreptococcus anaerobius*	Upregulates the expression of ALDH1A3, which utilises retinal as a substrate to generate NADH, essential for FSP1‐mediated synthesis of reduced coenzyme Q10	CRC	/	Cell lines (HT29, MC38, HEK293T, HT1080 and 786‐O), mouse models, human faecal samples	^59^
SBAs	/	Gut microbiota, particularly *Clostridium scindens*	Impair NKT cell accumulation by reducing CXCL16 expression of liver sinusoidal endothelial cells	Liver cancer	/	Mouse models	^60^
LCA	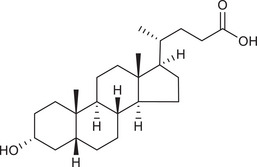	Gut microbiota	Stimulates IL‐8 expression by activating Erk1/2 and suppressing STAT3 activity	CRC	/	Cell lines (HCT116)	^61^
		Gut microbiota	Promotes miR21 expression via ERK1/2 activation and STAT3 inhibition	CRC	/	Cell lines (HCT116)	^62^
DCA	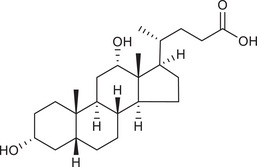	Gut microbiota	Causes DNA damage and NF‐κB activation through the induction of ROS	Oesophageal adenocarcinoma	At doses of 100 microM and higher	Cell lines (OE33)	^63^
		Gut microbiota	Suppresses CD8^+^ T cell by targeting plasma membrane Ca^2+^ ATPase to inhibit Ca^2+^‐nuclear factor of activated T cells signalling	CRC	/	Cell lines (HEK293T, HEK293F, MC38, Jurkat and B16‐OVA), mouse models, human tumour tissues and faecal samples	^64^
		Gut microbiota	Promotes vasculogenic mimicry formation and epithelial–mesenchymal transition via VEGFR2 activation	CRC	High‐fat diet	Cell lines (HCT116), mouse models, clinical sample collection and trial	^65^
UDCA	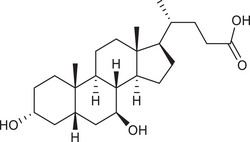	Gut microbiota	The concrete mechanism remains unclear because the conclusion is from clinical trial statistics	Colorectal neoplasia in patients with ulcerative colitis and primary sclerosing cholangitis	High‐dose UDAC	No experiment, clinical trial	^66^
TMAO	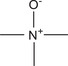	Gut microbiota	Induces the secretion of VEGFA from tumours to promote the proliferation and angiogenesis of cancer cells	CRC	/	Cell lines (HCT116), mouse models	^67^
Formate	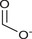	Gut *Fusobacterium nucleatum*	Triggers AhR signal transduction to amplify Th17 cells, increases tumour stemness and drives tumour invasion	CRC	At concentrations of 10 mM	Cell lines (HCT116, HT‐29 and Caco‐2), mouse models, human faecal sample, primary organoid	^68^
Succinic acid	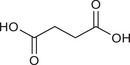	Gut *Fusobacterium nucleatum*	Inhibits the cGAS‐IFN‐β pathway to limit CD8^+^ T cell trafficking to the TME	CRC	/	Cell lines (HT‐29, LoVo, CT‐26 and MC38), mouse models, human faecal and serum samples	^4^
Lactic acid	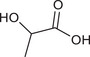	Intratumoural *Lactobacillus iners* (L‐lactate)	Metabolic rewiring or alterations in multiple metabolic pathways in tumours, such as Warburg effect, glycolysis, glutamate metabolism and galactose metabolism	Cervical cancer	/	Primary cells, cell lines (HeLa, SiHa, CaSki), patient‐derived organoids, swabs and cytobrush samples and blood samples	^69^
Urolithin A	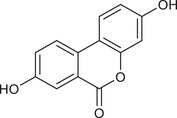	Gut microbiota	Has the potential to interfere with taxane chemotherapy by reducing tubulin polymerisation while inhibiting P‐glycoprotein drug efflux	Castration‐resistant prostate cancer	/	Cell lines (22Rv1, PC‐3 and C4‐2), mouse models	^70^

Abbreviations: ACACA, acetyl‐CoA carboxylase alpha; ACSS2, acyl‐CoA synthetase short‐chain family member 2; AhR, aryl hydrocarbon receptor; ALDH1A3, aldehyde dehydrogenase 1 family member A3; Arg1, arginase‐1; cGAS, cyclic GMP–AMP synthase; CXCL, C‐X‐C motif chemokine ligand; DC, dendritic cell; DCA, deoxycholic acid; ERK, extracellular regulated kinase; FASN, fatty acid synthase; FSP1, ferroptosis‐suppressor protein 1; HDACs, histone deacetylases; H3K27, histone H3 lysine 27; IAA, indole acetic acid; ICOS, inducible co‐stimulator; IDA, trans‐3‐indolacrylic acid; IFN, interferon; IGF1, insulin‐like growth factor 1; IL, interleukin; LCA, lithocholic acid; LDHA, lactate dehydrogenase A; LPS, lipopolysaccharide; MAPK, mitogen‐activated protein kinase; MCT1, monocarboxylate transporter 1; NAFLD‐HCC, non‐alcoholic fatty liver disease‐related hepatocellular carcinoma; NKT, natural killer T; PD‐L1, programmed cell death‐ligand 1; PI3K, phosphatidylinositol‐3‐kinase; ROS, reactive oxygen species; SBAs, secondary BAs; SCFAs, short‐chain fatty acids; STAT3, signal transducers and activators of transcription 3; TAMs, tumour‐associated macrophages; Th, helper T cell; TMAO, trimethylamine‐N‐oxide; TME, tumour microenvironment; UDCA, ursodeoxycholic acid; UDP‐GlcNAc, uridine diphospho‐N‐acetylglucosamine; VEGFA, vascular endothelial growth factor A; VEGFR, vascular endothelial growth factor receptor.

### 2.1 Gut microbial metabolites

#### 2.1.1 Short‐chain fatty acids

**FIGURE 3:**
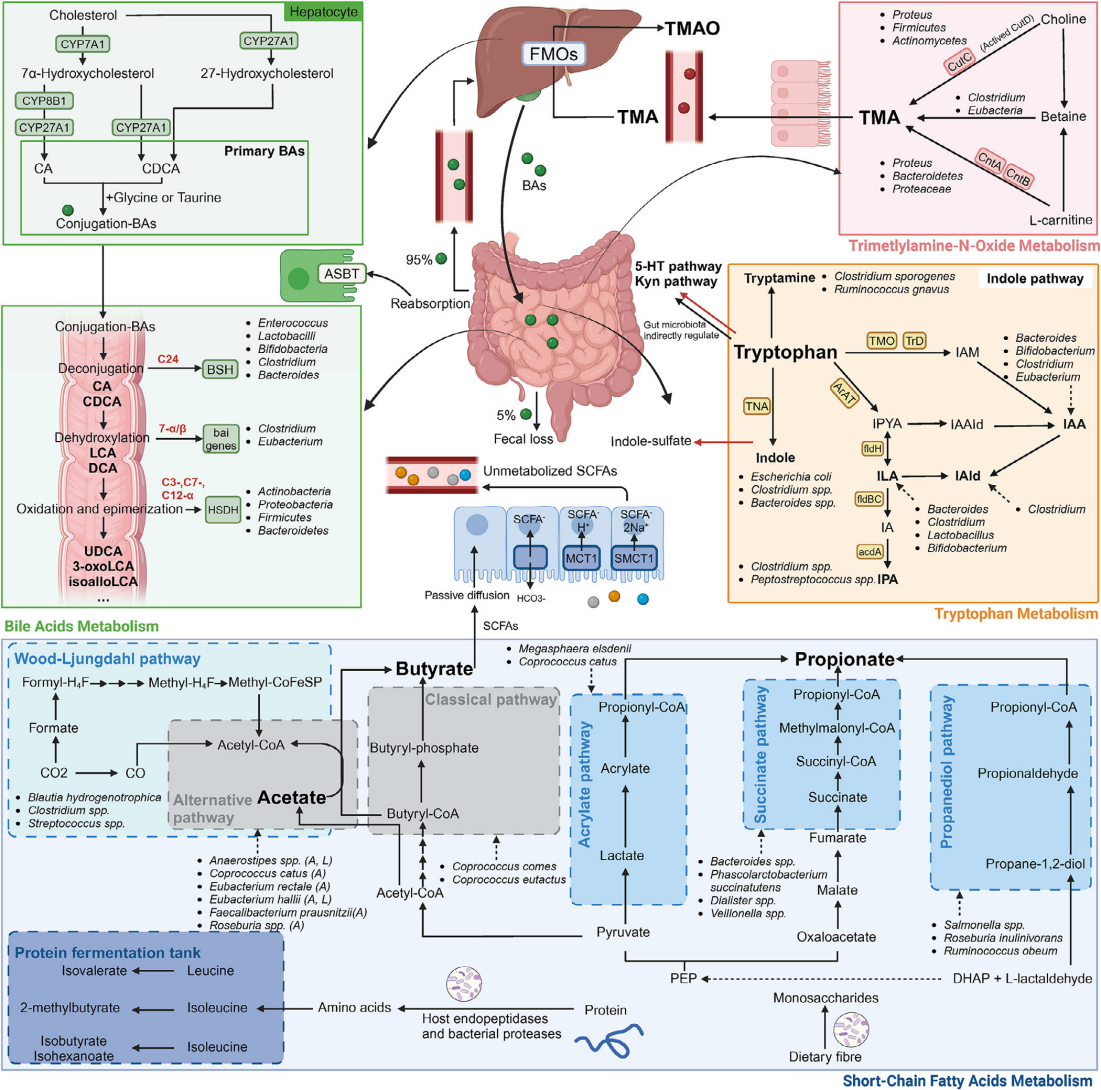
The production processes of several main types of microbial metabolites. (A) Short‐chain fatty acids metabolism. Most intestinal bacteria produce acetate through acetyl‐CoA derived from pyruvate, while some intestinal bacteria synthesise acetate by fixing CO_2_ via the Wood–Ljungdahl pathway; propionate has three synthetic paths including the succinate pathway, the acrylic ester pathway and the propylene glycol pathway; butyryl‐CoA can be converted to butyrate via the so‐called classical pathway or the acetate‐CoA transferase pathway; protein fermentation mainly produces branched‐chain fatty acids. (B) Tryptophan metabolism. The main introduction was about the indole pathway which gut microbiota directly involve in. (C) Bile acids metabolism. The process of hepatocytes metabolising cholesterol to produce primary bile acids and the gut microbiota metabolising primary bile acids to produce secondary bile acids is shown. (D) Trimethylamine‐N‐oxide metabolism. Gut microbiota converts choline, L‐carnitine and betaine into TMA in the colon, which then enters the liver via the portal vein circulation and is oxidised into TMAO. acdA, acyl‐CoA dehydrogenase; ArAT, aromatic amino acid aminotransferase; ASBT, apical sodium‐dependent bile acid transporter; BAs, bile acids; BSH, bile salt hydrolases; CA, cholic acid; CDCA, chenodeoxycholic acid; CntA/CntB, carnitine monooxygenase; CutC/CutD, choline trimethylamine‐lyase; CYP7A1, cholesterol 7α‐hydroxylase; CYP27A1, sterol 27‐hydroxylase; CYP8B1, sterol 12α‐hydroxylase; DCA, deoxycholic acid; DHAP, dihydroxyacetone phosphate; fldBC, phenyllactate dehydratase; fldH, phenyllactate dehydrogenase; FMOs, flavin monooxygenases; HSDH, hydroxysteroid dehydrogenases; IA, indole acrylic acid; IAA, indole acetic acid; IAAld, indole‐3‐acetaldehyde; IAld, indole‐3‐aldehyde; IAM, indole‐3‐acetamide; ILA, indole‐3‐lactic acid; IPA, indole‐3‐propionic acid; IPYA, indole‐3‐pyruvate; LCA, lithocholic acid; MCT1, monocarboxylate transporter 1; PEP, phosphoenolpyruvate; SCFAs, short‐chain fatty acids; SMCT1, sodium‐coupled monocarboxylate transporter 1; TMA, trimethylamine; TMAO, trimethylamine‐N‐oxide; TMO, tryptophan 2‐monooxygenase; TNA, tryptophanase; TrD, tryptophan decarboxylase; UDCA, ursodeoxycholic acid.

SCFAs are saturated fatty acids with chain lengths of two to six carbon atoms and represent the most abundant intestinal microbial metabolites. Acetate, propionate and butyrate account for over 90% of total intestinal SCFAs, mainly deriving from the fermentation of dietary fibre mediated by the gut microbiota. *Akkermansia muciniphila* and its subtypes can also produce SCFAs through degrading mucin in the intestine.^71^ Following SCFAs production, 95% of SCFAs are absorbed by colonic epithelial cells via passive diffusion, 1:1 exchange with HCO3^−^ or transporting via monocarboxylate transporter 1 (MCT1) and sodium‐coupled monocarboxylate transporter 1 (SMCT1) channels. Butyrate serves as preferred energy source for colonocytes, while other unmetabolised SCFAs are transported via the portal vein into systemic circulation and peripheral tissues (Figure 3). As signalling molecules between the commensal microbiota and the host, SCFAs regulate host physiology and pathology mainly through inhibiting the activity of histone deacetylases (HDACs) and activating signalling cascades mediated by various G protein‐coupled receptors (GPCRs), including GPR41, GPR43 and GPR109A.^72^ Activation of these GPCRs inhibits cAMP‐dependent signalling pathways while activating alternative signalling pathways, such as mTOR signalling.^73^

##### Butyrate

Research on the role of SCFAs in tumours has thus far focused on butyrate, which is considered a tumour suppressor in a myriad of different cancer types, particularly colorectal cancer (CRC). Compared to healthy individuals, the abundance of butyrate‐producing bacteria in the colons of CRC patients is significantly decreased, further supporting the role of butyrate in CRC.^74^ Mechanistically, butyrate acts as a signalling molecule to alter multiple pathways that control cell proliferation, apoptosis, the epithelial–mesenchymal transition (EMT) and other malignant processes. Butyrate suppresses intestinal tumour cell proliferation by inhibiting calcineurin‐mediated activation of the nuclear factor of activated T cells 3 (NFATc3) transcription factor.^9^ Butyrate can regulate the expression of cyclin E1, which is crucial for cancer cell cycle regulation.^75^ Butyrate can also reduce vascular endothelial growth factor (VEGF) secretion and inhibit tumour progression through its inhibition of HDACs.^10^, ^76^ Meanwhile, butyrate can enhance nuclear factor kappa B (NF‐κB) signalling activation mediated by Toll‐like receptor 4 (TLR4) in colon cancer cells, thereby strengthening anti‐tumour innate immunity.^11^ Moreover, butyrate can reduce the expression of P53, P21 and β‐catenin to inhibit CRC cell proliferation.^12^, ^13^ Not only that, SCFAs can also induce CRC cell apoptosis by downregulating the expression of phosphatidylinositol‐3‐kinase (PI3K)/serine/threonine kinase (Akt), increasing cytochrome c release, and subsequently activating caspase‐9 and caspase‐3.^6^ Furthermore, butyrate can inhibit xCT‐dependent glutathione synthesis through the inhibition of class I HDAC activity, thus promoting ferroptosis induced by oxaliplatin.^14^

Butyrate can reshape the tumour immune microenvironment to alter tumour development. For instance, butyrate can suppress IL‐27 production by binding to GPR43 expressed by dendritic cells (DCs), which can reverse CD8^+^ T cell exhaustion.^7^ Butyrate further increases histone 3 lysine 27 (H3K27) acetylation to modulate T cell receptor signalling in cytotoxic CD8^+^ T cells, thereby boosting anti‐PD‐1 efficacy.^15^ *Roseburia intestinalis*‐derived butyrate activates cytotoxic CD8^+^ T cells by directly binding to TLR5 and activating NF‐κB signalling, which further increases anti‐PD‐1 therapy efficacy.^16^ Meanwhile, pentanoate and butyrate can act on HDACs to increase the anti‐tumour activity of CD8^+^ T cells through metabolic and epigenetic reprogramming, leading to increased production of effector molecules such as CD25, interferon‐γ (IFN‐γ) and tumour necrosis factor‐α (TNF‐α), which increased the efficacy of adoptive immunotherapy in melanoma and pancreatic cancer mouse models.^8^ Butyrate can also suppress the expression of the immunosuppressive factors PD‐L1 and IL‐10 in tumour‐associated macrophages in gastric cancer.^17^ Moreover, butyrate can enhance the response to immune checkpoint blockade (ICB) in CRC patients by triggering the expression of DNA binding 2 (ID2) inhibitor, which regulates IL‐12 signalling to promote the proliferation and anti‐tumour effects of CD8^+^ T cells.^18^ Furthermore, supplementing butyrate and the butyrate‐producing bacterium *Prevotella loescheii* can polarise colonic macrophages away from the M1‐like phenotype and downregulate the expression of the proinflammatory factors TNF‐α and IL‐1β, ultimately alleviating the immune‐related adverse events (irAEs) related to immunotherapy.^77^ Patients with long‐standing and poorly controlled inflammatory bowel disease (IBD) are at increased risk of colitis‐associated CRC.^78^ In addition to regulating immune cell responses, butyrate can suppress colonic inflammation and inhibit colorectal carcinogenesis. Butyrate can bind to GPR109a on DCs and macrophages to regulate CD4^+^ T cell differentiation, resulting in an increase in regulatory T cells (Tregs) and IL‐10‐producing CD4^+^ T cells and a reduction in Th17 cells.^79^, ^80^

However, recent studies have proposed that butyrate has a pro‐oncogenic role as well. Butyrate is capable of inducing hyperproliferation^48^ and cellular senescence^49^ in intestinal epithelial cells, which is implicated in tumourigenesis. SCFAs can also stimulate the production of insulin‐like growth factor 1 (IGF1) to promote the growth of prostate cancer by activating the MAPK and PI3K signalling.^47^ Moreover, butyrate can undermine the radiotherapy‐induced antigen presentation to DCs, thereby impairing the tumour‐killing effect mediated by CD8^+^ T cells.^51^ Notably, a clinical study involving 38 patients treated with CTLA‐4 monoclonal antibody (mAb) ipilimumab revealed that elevated levels of peripheral butyrate were negatively correlated with treatment response, potentially by impairing anti‐tumour immunity.^52^ Moreover, excess butyrate was found in non‐alcoholic fatty liver disease‐related hepatocellular carcinoma (NAFLD‐HCC) patients, and the excess butyrate led to the expansion of Tregs but a reduction in CD8^+^ T cells, thus disrupting the anti‐tumour immune response.^53^

##### Acetate

Acetate is the predominant SCFA in the peripheral circulation. Acetate can activate GPR43 to promote the resolution of inflammation in intestinal mouse models to suppress the risk of colitis‐associated CRC.^81^ Gut microbiota‐derived acetate can modulate type 3 innate lymphoid cell function by inhibiting HDAC activity and boost anti‐PD1 efficacy in hepatocellular carcinoma (HCC) mouse models.^20^ Additionally, acetate can upregulate glutamine and UDP‐GlcNAc levels and enhance protein O‐GlcNAcylation, while hyper‐O‐GlcNAcylation of eukaryotic elongation factor 1A1 promotes cell proliferation.^54^ Due to its simple chemical structure and pivotal role in metabolic pathways, acetate can also serve as a nutrient source and play a role in tumour progression. The metabolic connection between acetate and acetyl‐CoA is essential to various physiological processes within tumours.^82^ For instance, CD8^+^ memory T cells can take up acetate and utilise it to synthesise acetyl‐CoA, thereby promoting glycolysis, which can potentially enhance the anti‐tumour immune response.^21^ Not only that, under energy deficiency conditions, acetate can be taken up by CD8^+^ T cells and NK cells in the tumour microenvironment (TME), and then converted into acetyl‐CoA by acyl‐CoA synthetase short‐chain family member 2 (ACSS2), thereby promoting immune cells proliferation and anti‐tumour immune function.^22^ Conversely, under hypoxic conditions, malnourished tumour cells can exploit acetate as a carbon source to replenish intracellular acetyl‐CoA levels for catabolic and anabolic needs, thus promoting tumour growth.^55^ Moreover, tumour cells can take up acetate and convert it to acetyl‐CoA through ACSS2, resulting in elevated c‐Myc acetylation, which in turn reprograms tumour metabolism and enhances PD‐L1 expression and immune evasion.^56^

##### Propionate

Propionate can activate GPR and, to a lesser extent, act as an HDAC inhibitor, thereby exerting anti‐tumour effects.^23^ Similar to butyrate, propionate can inhibit inflammation and help maintain intestinal homeostasis. Propionate can promote intestinal goblet cell differentiation through GPR41^83^ and directly inhibit γδ T cells from producing interleukin‐17 (IL‐17) and IL‐22 in an HDAC‐dependent manner.^84^ Propionate can also serve as a natural ligand for two known GPCRs, GPR41 and GPR43, which inhibit the mitogen‐activated protein kinase (MAPK) signalling pathway and Hippo‐Yap, respectively, thereby inhibiting the invasive and metastatic capacity of breast cancer cells.^24^ Moreover, *Akkermansia muciniphila* protects the intestine from irradiation‐induced injury by secreting propionate, which can bind to GPR43 on the intestinal epithelia surface, thereby increasing the expression of tight junction proteins and the level of mucins.^85^ However, notably, another study also demonstrated that a high concentration of propionate can result in the accumulation of metabolic by‐products such as methylmalonic acid, which may promote the invasiveness of breast cancer and lung cancer cells.^57^

#### 2.1.2 Microbial tryptophan metabolites

Tryptophan (Trp), an essential aromatic amino acid, is involved in three pathways: the kynurenine (Kyn) pathway, the 5‐hydroxytryptamine (5‐HT) pathway and the indole pathway. These pathways involve multiple organs, such as the liver, brain and intestines, thereby influencing various physiological and pathological processes. The gut microbiota, such as *Anaerostipes*, *Bacteroides*, *Clostridium*, *Bifidobacterium* and *Lactobacillus*, directly converts Trp into indole and its derivatives, which is known as the indole pathway. Numerous indole derivatives can act as ligands for the aryl hydrocarbon receptor (AhR), a ligand‐responsive transcription factor with diverse physiological functions. Moreover, the gut microbiota also plays an indirect regulatory role in the Kyn pathway and the 5‐HT pathway. It is pivotal in activating indoleamine‐2,3‐dioxygenase 1 (IDO1), the initial rate‐limiting enzyme that catalyses the transformation of Trp to Kyn, and the production of homologous metabolic enzymes. Moreover, the gut microbiota may influence the production of intestinal 5‐HT in enterochromaffin cells, but the underlying mechanism is not yet fully understood (Figure 3).^86^ Microbial Trp metabolites can play an essential role in malignancy in an AhR‐dependent or AhR‐independent manner. Next, the effects of microbial metabolites derived from tryptophan in tumours are categorically introduced according to their mechanisms of action.

##### AhR‐dependent metabolites

Bacteria‐derived Trp metabolites such as trans‐3‐indolacrylic acid (IDA), indole‐3‐aldehyde (I3A), indole‐3‐carboxylic acid (ICA) and indole acetic acid (IAA) are defined as AhR ligands that play important roles in cellular homeostasis, host immunity and tumour progression, suggesting that AhR acts as a mediator between Trp‐metabolising bacteria and the host.

Indole and its derivatives derived from *Lactobacillus reuteri* activate AhR and upregulate the expression of sterol regulatory element‐binding protein 2 (SREBP2), thereby inhibiting the development of liver cancer.^25^ In addition, indolepropionic acid and indoxylsulfate activate AhR to induce oxidative stress, leading to the inhibition of breast cancer cell growth.^87^, ^88^ Importantly, the immunoregulatory role of tryptophan metabolite‐mediated AhR signalling in anti‐tumour effects has recently been revealed. ICA, a *Lactobacillus gallinarum*‐derived metabolite, suppresses the key enzyme responsible for producing Kyn, competitively inhibits Kyn‐induced AhR activation, and ultimately enhances CD8^+^ T cell function to boost anti‐PD1 efficacy in CRC patients.^28^ Conversely, dietary tryptophan can be metabolised into indole by *Lactobacillus*, which activates AhR in tumour‐associated macrophages (TAMs), reducing the ability of CD8^+^ T cells to suppress anti‐tumour immunity and ultimately promoting pancreatic ductal adenocarcinoma development.^58^ Moreover, IDA derived from *Peptostreptococcus anaerobius* has been reported to transcriptionally upregulate the expression of aldehyde dehydrogenase 1 family member A3 (ALDH1A3) in an AhR‐dependent manner, contributing to the malignant progression of CRC by inhibiting ferroptosis.^59^ AhR signals under different metabolic conditions have different effects on tumours, thus increasing the complexity of the ‘tryptophan metabolite‐AhR’ signalling pathway.

##### AhR‐independent metabolites

The anti‐tumour effects of some microbial Trp metabolites persisted despite the use of an AhR inhibitor, indicating they act through an AhR‐independent mechanism. In vitro and in vivo studies have demonstrated that 3‐indolepropionic acid (IPA) can potentiate the anti‐tumour effect of γδ T cells by increasing their cytotoxic capacity and stimulating the release of granzyme B and perforin.^29^ IPA can also modulate the stemness of CD8^+^ T cells, thereby improving pan‐cancer responses to ICB; this improvement can be seen in melanoma, breast cancer and CRC patients.^30^ Furthermore, both *Lactobacillus reuteri* and *Lactobacillus plantarum* enhance anti‐tumour immunity in CRC by inhibiting Th17 cell differentiation and promoting DC function through indole‐3‐lactic acid, respectively.^31^, ^32^ Additionally, microbial Trp metabolites can influence cancer cell proliferation by affecting oxidative stress. For instance, indole‐3‐acetic acid (3‐IAA) can be converted into a toxic molecule by myeloperoxidase in neutrophils, resulting in the accumulation of reactive oxygen species (ROS) within cancer cells, impairing autophagy and enhancing the efficacy of chemotherapeutics in pancreatic cancer patients.^26^

#### 2.1.3 Bile acids

Bile acids (BAs) metabolism involves two parts: hepatocytes synthesis of primary BAs and their conversion into secondary BAs (SBAs) mediated by the intestinal microbiota (Figure 3). In hepatocytes, cholesterol is converted into primary BAs, including cholic acid (CA) and chenodeoxycholic acid (CDCA), and their conjugates with glycine or taurine. In the colon, primary BAs are converted into SBAs through a series of biotransformation reactions, including deconjugation mediated by bile salt hydrolases (BSHs), 7α/β‐dehydroxylation mediated by BA‐inducible (bai genes and oxidation and epimerisation mediated by hydroxysteroid dehydrogenases (HSDHs). As endogenous ligands that can activate a series of receptors, including G‐protein‐coupled bile acid receptor 1 (GPBAR1; also known as TGR5) and farnesoid X receptor (FXR), SBAs participate in the regulation of various physiological functions and immune responses in the host.^89^

##### DCA and LCA

As a highly potent antibacterial BAs, deoxycholic acid (DCA) can significantly suppress the growth of beneficial gut microbes, including *Lactobacillus* and *Bifidobacterium*, suggesting that SBAs may promote cancer development by regulating the composition of the microbiota.^90^ A pre‐clinical study showed that DCA treatment reduces the abundance of *Lactobacillus gasseri* and multiple butyrate‐producing bacteria, such as *Clostridium leptum*, *Lachnospiraceae bacterium* and *Eubacterium coprostanoligenes*, in mice, thereby accelerating intestinal inflammation and tumour progression.^91^ As candidate carcinogens, SBAs generate cellular ROS that cause DNA damage and genomic instability.^63^, ^92^ Moreover, numerous studies have shown that SBAs can reshape the TME and promote tumourigenesis. For instance, SBAs impair natural killer T (NKT) cell activity and suppress anti‐tumour immunity in the liver, thereby promoting tumour growth.^60^ DCA can suppress CD8^+^ T cell anti‐tumour functions by targeting plasma membrane Ca^2+^ ATPase (PMCA) to inhibit Ca^2+^‐NFAT2 signalling, thereby promoting CRC growth.^64^ Moreover, lithocholic acid (LCA) can stimulate IL‐8 expression in human CRC cells, thereby promoting endothelial cell proliferation and ultimately contributing to a poor prognosis.^61^ In addition, SBAs regulate the activation and signalling of a series of pathways, such as the STAT3,^62^, ^93^ WNT/β‐catenin,^94^ and NF‐kB pathways,^36^ to promote tumour development. DCA promotes vasculogenic mimicry (VM) formation and EMT through VEGFR2 activation, which further exacerbates intestinal carcinogenesis.^65^

However, recent studies have shown that DCA and LCA can also act as tumour suppressors. One study suggested that LCA played a potential therapeutic role in breast cancer cells through the reversion of lipid metabolism deregulation to induce apoptosis.^33^ Moreover, DCA induces mucin 2 (MUC2) expression and suppresses tumour invasion in gastric carcinomas,^34^ oesophageal adenocarcinoma,^95^ and colon carcinoma.^96^ Furthermore, DCA can inhibit cell proliferation by decreasing miR‐92b‐3p expression in a m6A‐dependent manner, ultimately inactivating the PI3K/AKT signalling pathway to suppress the progression of gallbladder cancer (GBC).^35^

In addition, newly discovered secondary BA derivatives, such as 3‐oxoLCA, isoalloLCA and iso‐DCA, also possess immunomodulatory properties. For example, administration of 3‐oxoLCA and isoalloLCA to mice reduced Th17 cell differentiation by directly binding to the key transcription factor retinoid‐related orphan receptor γt (RORγt), increased Treg differentiation through the production of mitochondrial ROS and increased Foxp3 expression in the intestinal lamina propria.^97^ Moreover, iso‐DCA can also increase the induction of Foxp3 by acting on DCs, thus promoting the production of Treg cells.^98^

##### UDCA

Unlike DCA and LCA, ursodeoxycholic acid (UDCA) is a type of hydrophilic BA generally considered a protective factor due to its ability to prevent cholestasis and protect hepatocytes from oxidative damage.^99^ Moreover, UDCA can inhibit NF‐ĸB signalling^36^ and suppress cyclooxygenase‐2 (Cox‐2) expression^37^ to stop the progression of colon cancer. UDCA also enhances anti‐tumour immunity by degrading transforming growth factor β (TGF‐β) and suppressing the differentiation and activation of Tregs in mice.^38^ However, high‐dose UDCA has been reported to have cancer‐promoting effects, but the underlying mechanism remains unclear and requires further thorough investigation.^66^

#### 2.1.4 Trimethylamine‐N‐oxide

Trimethylamine‐N‐oxide (TMAO), an N‐oxide of trimethylamine (TMA), is a bioactive metabolite derived directly from dietary intake and indirectly from gut microbial metabolism. The latter one is involved in the conversion from the choline, L‐carnitine and betaine consumed in food into TMA in the colon, which enters the liver via portal vein circulation and is oxidised into TMAO by flavin monooxygenases (FMOs^100^; Figure 3). Recently, emerging evidence has underscored the dual immunomodulatory effect of TMAO on cancer.

A prospective cohort study suggested that increased circulating TMAO levels are associated with an increased risk of CRC.^101^ Genome‐wide system analysis revealed that TMAO is closely genetically associated with CRC, indicating the crucial role of TMAO in linking gut microbial metabolism to CRC.^102^ Mechanistic studies demonstrate that TMAO primarily promotes CRC progression by inducing inflammation and oxidative damage.^103^ Moreover, TMAO can also induce tumour cell proliferation and colon cancer progression by promoting angiogenesis.^67^ However, some studies have also shown that TMAO promotes anti‐tumour immunity. For example, TMAO can enhance the activity of the type I interferon pathway and induce an immunostimulatory TAM phenotype to activate effector T cells, thereby sensitising pancreatic ductal adenocarcinoma to ICB therapy.^39^

#### 2.1.5 Inosine

Inosine is a purine metabolite produced by the gut microbiota of *Akkermansia muciniphila* and *Bifidobacterium pseudolongum*.^104^ In CRC, bladder cancer and melanoma mouse models, administering inosine derived from *Bifidobacterium pseudolongum* can enhance immune responses and the therapeutic effects of ICB. Mechanistically, inosine acts on the adenosine A2A receptor expressed on T cells under co‐stimulation to promote Th1 cell differentiation.^41^ In contrast, inosine has also been found to suppress Th1 differentiation in the absence of IFN‐γ, suggesting that the efficient anti‐tumour effect of inosine on tumours requires sufficient co‐stimulation.^105^

#### 2.1.6 Formate

In mammals, formate serves as a 1C unit and is closely related to the metabolism of many substances.^106^ For example, endogenous formate has been shown to promote the invasion of glioblastoma cells by influencing lipid metabolism in vitro.^107^ *F. nucleatum*‐derived formate can enhance AhR signalling and amplify the activity of Th17 cells, thereby promoting CRC invasion and metastasis.^68^ These studies indicated that formate derived from the gut microbiota is a cancer‐related metabolite.

#### 2.1.7 Succinic acid

In addition to being synthesised in human cells, succinic acid is also considered a metabolic product of the gut microbiota in the intestinal cavity and faeces, primarily derived from *Bacteroidetes, Bifidobacteria, Veillonella* and *Prevotella*.^108^ Research has shown that succinic acid produced by *F. nucleatum* inhibits the cyclic GMP—AMP synthase (cGAS)‐IFN‐β pathway, thereby reducing the levels of the Th1‐type chemokines CCL5 and CXCL10 in tumours. This reduction, in turn, inhibits the anti‐tumour response by limiting the transport of CD8^+^ T cells to the TME, ultimately leading to immunotherapy resistance in patients with CRC.^4^

#### 2.1.8 Lactic acid

Lactic acid can be classified into two types: L‐lactic acid and D‐lactic acid, with the latter exclusively produced by microorganisms, such as *Lactobacillus iners* and *Bifidobacterium*. D‐Lactic acid has been reported to transform M2‐TAMs towards the M1 phenotype, thereby remodelling the immunosuppressive TME and becoming a potential target for promoting immunotherapy response in HCC patients.^42^ The *Lactobacillus iners* metabolite lactate can activate the Wnt pathway via the lactate‐Gpr81 complex; this activation leads to elevated core fucosylation in epidermal cells, thereby suppressing the proliferation and migration of cervical cancer cells.^43^

#### 2.1.9 Urolithin

Urolithin, which is converted from ellagitannin by intestinal *Gordonibacter* and *Ellagibacter*, confers various benefits to the host. Mechanistically, urolithin A (UA) can inhibit intestinal inflammation by suppressing NF‐κB pathway activity and preserving DNA integrity.^109^ Additionally, UA activates AhR‐Nrf2‐dependent pathways to upregulate the expression of epithelial tight junction proteins, thus helping maintain the integrity of the intestinal barrier and reducing intestinal inflammation.^110^ Urolithins also exert anti‐tumour effects by modulating various signalling pathways. For example, urolithin B (UB) can inhibit the proliferation of HCC cells in vitro and in vivo by deactivating Wnt/β‐catenin signalling.^111^ UA can also block the phosphorylation of AKT and p70S6K, thereby successfully suppressing pancreatic cancer growth.^44^ Furthermore, UA induces senescence‐associated β‐galactosidase activity, resulting in p53‐dependent cellular senescence in CRC cells.^45^ Moreover, UA triggers mitophagy in CD8^+^ T cells by upregulating Wnt signalling and promoting the formation of T memory stem cells to boost anti‐tumour immunity.^46^ However, as with many of the microbial metabolites mentioned above, urolithin also has paradoxical functions in tumours. UA has the potential to interfere with taxane treatment of castration‐resistant prostate cancer by reducing its tubulin polymerising effect.^70^

#### 2.1.10 Other associated gut microbial metabolites

With the optimisation of identification and isolation systems, an increasing number of gut microbial metabolites implicated in regulating tumour biology have been identified. Reuterin produced by *Lactobacillus reuteri* induces selective protein oxidation and suppresses ribosomal biogenesis and protein translation to restrict colon tumour growth.^112^ Moreover, both the exopolysaccharides and ferrichrome derived from *Lactobacillus* can induce intrinsic apoptosis of tumour cells via the ER stress‐responsive signalling pathway.^113^, ^114^ Dysfunctional circadian clock can affect the gut microbiota, leading to the accumulation of a gut microbial metabolite, taurocholic acid, which promotes glycolysis of myeloid‐derived suppressor cells (MDSCs) epigenetically and induces them to aggregate in the lungs, making CRC more prone to lung metastasis.^115^ The identification of these previously unrecognised microbial metabolites with regulatory functions underscores the need for further exploring this emerging field.

### 2.2 Intratumoural microbial metabolites

**FIGURE 4:**
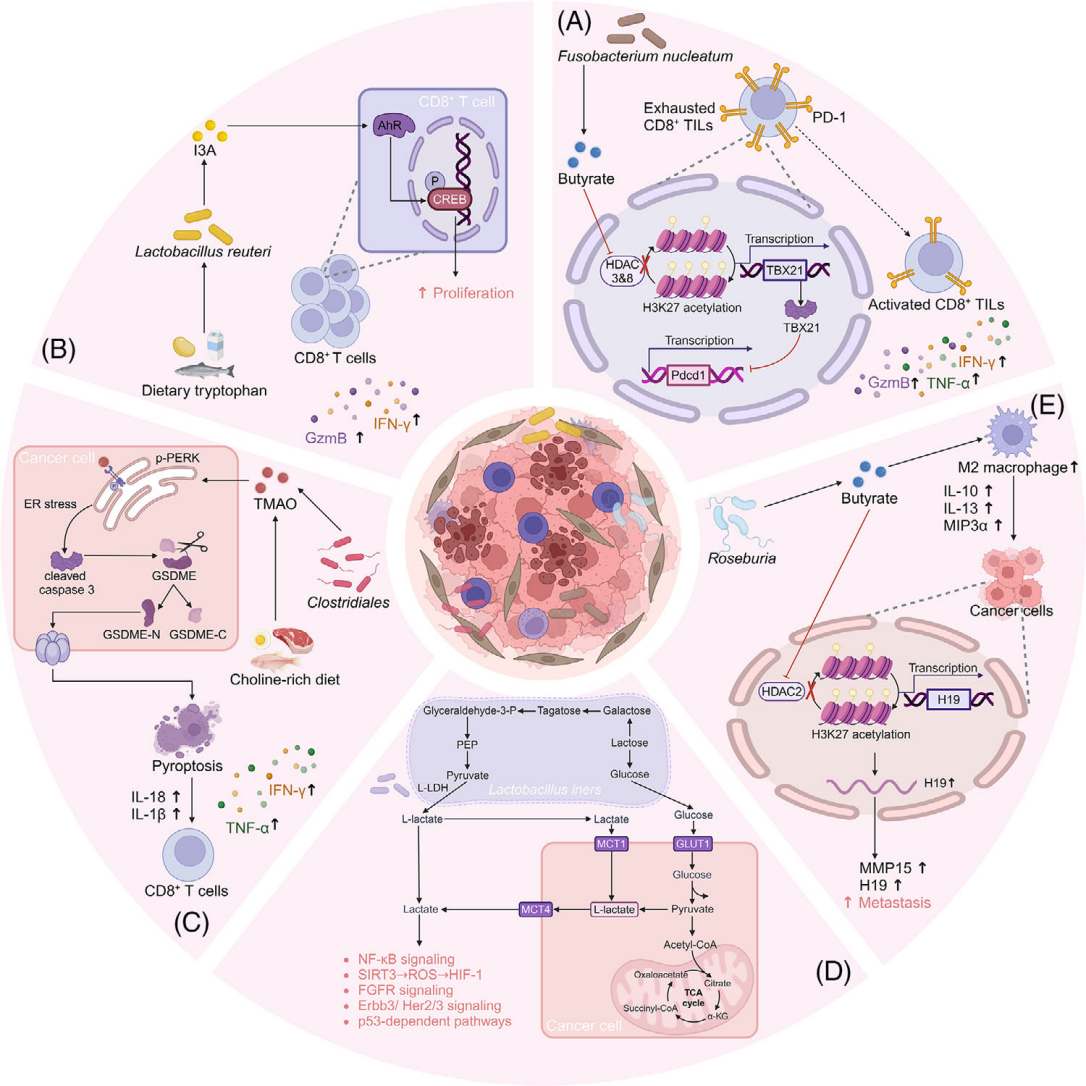
Functional mechanisms of intratumoural microbial metabolites. (A) Intratumoural *Fusobacterium nucleatum*‐derived butyrate suppresses elevated PD‐1 expression to inhibit CD8^+^ T cell exhaustion. (B) Intratumoural *Lactobacillus reuteri*‐derived I3A strengthens CD8^+^ T cell function by activating AhR. (C) Intratumoural *Clostridiales*‐derived TMAO induces pyroptosis in triple‐negative breast cancer cells and thus promotes CD8^+^ T cell‐mediated anti‐tumour immunity. (D) Tumour‐resident *Lactobacillus iners*‐derived L‐lactate induces metabolic reprogramming in cervical cancer cells. (E) Intratumoural *Roseburia*‐derived butyrate enhance the metastatic capabilities of tumour cells and facilitates M2 macrophage polarisation. AhR, aryl hydrocarbon receptor; CREB, cAMP‐response element binding protein; Erbb3, epidermal growth factor receptor 3; ER stress, endoplasmic reticulum stress; FGFR, fibroblast growth factor receptor; GLUT1, glucose transporter type 1; GSDME, gasderminE; Gzm B, granzyme B; HDACs, histone deacetylases; HIF, hypoxia inducible factor; IFN‐γ, interferon‐γ; IL, interleukin; I3A, indole‐3‐aldehyde; L‐LDH, L‐lactate dehydrogenase; MCT, monocarboxylate transporter; MH3K27, histone H3 lysine 27; MP15, matrix metalloproteinase 15; µIP3α, macrophage inflammatory protein 3α; NF‐κB, nuclear factor kappa B; PD‐1, programmed cell death protein 1; PEP, phosphoenolpyruvate; PERK, PKR‐like endoplasmic reticulum kinase; ROS, reactive oxygen species; SIRT, sirtuins; TBX21, T‐box transcription factor 21; TCA cycle, tricarboxylic acid cycle; TIL, tumour infiltrating lymphocyte; TMAO, trimethylamine‐N‐oxide; TNF‐α, tumour necrosis factor‐α.

Research on intratumoural microbial metabolites remains scarce compared to that focused on gut‐derived microbial metabolites. However, recent studies have consistently highlighted the distinctive functional roles of intratumoural microbial metabolites in situ, underscoring the necessity for further exploration in this emerging field (Figure 4). Butyrate produced by intratumoural *F. nucleatum* inhibits HDAC3/8 activity in CD8^+^ T cells, leading to increased H3K27 acetylation at the TBX21 promoter, which suppresses PD‐1 expression and enhances anti‐PD‐1 therapeutic efficacy in CRC.^19^ Intratumoural *Lactobacillus reuteri* can convert dietary tryptophan into I3A to strengthen CD8^+^ T cell function by activating AhR in melanoma patients, thus improving the efficacy of ICB treatment.^27^ Additionally, intratumoural *Clostridiales*‐derived TMAO induces pyroptosis in triple‐negative breast cancer cells by activating the PERK on the endoplasmic reticulum membrane and thus promotes CD8^+^ T cell‐mediated anti‐tumour immunity, which can be enhanced by supplementing with choline.^40^

Consistent with gut microbial metabolites, specific intratumoural microbial metabolites also can conversely promote tumour progression. Through inhibiting HDAC2 binding to the H19 promoter, intratumoural *Roseburia*‐derived butyrate increases H3K27 acetylation and subsequently enhancing H19 expression, which promotes lung cancer cell progression. Meanwhile, butyrate facilitates M2 macrophage polarisation, accompanied by the secretion of various metastasis‐promoting cytokines, such as IL‐10, IL‐13 and MIP3a.^116^ Another study revealed that tumour‐resident *Lactobacillus iners* can produce L‐lactic acid, thereby inducing metabolic reprogramming and chemoradiation resistance in cervical cancer cells.^69^

## 3 CLINICAL APPLICATION POTENTIAL OF MICROBIAL METABOLITES

The microbiome has shown striking value as multifaceted biomarkers in cancer clinical applications.^117^ Given that changes in the metabolome can effectively mirror the functional status of microorganisms, profiling microbial metabolites may translate into non‐invasive biomarkers for cancer diagnosis, therapeutic efficacy assessment and prognostic prediction (Table 3). A blood‐based metabolomic signature, which includes three microbiome‐related metabolites, can predict the 5‐year risk of pancreatic cancer.^118^ Integrated analysis of targeted and untargeted serum metabolomics and faecal samples metagenome sequencing, a model based on 8 gut microbiome‐associated serum metabolites is identified to differentiate CRC patients and individuals with benign lesions as well as healthy individuals, which demonstrated a higher area under the curve (AUC) compared to traditional biomarker carcinoembryonic antigen (CEA).^119^ Another multi‐centre population study also identified a combination of 17 plasma metabolites that can accurately diagnose CRC (AUC = .848–.987).^120^ Notably, the combination of microbial and metabolite features has been proven in multiple studies to exhibit better diagnostic accuracy compared to their individual applications.^91^, ^121^, ^122^ In addition, differentially abundant microbial metabolites also have translational potential as non‐invasive biomarkers for efficacy prediction and prognostic assessment. The concentrations of SCFAs in faecal and plasma samples have demonstrated predictive value for cancer immunotherapy efficacy.^123^, ^124^ Moreover, the combination of gut microbiota and metabolites represents potential prognostic and predictive biomarkers for clinical response in biliary tract cancer patients undergoing anti‐PD‐1/PD‐L1 therapy.^125^, ^126^ In addition, the prognostic role of the gut microbiota in the clinical outcomes of patients undergoing primary CRC resection, with microbial metabolites serving as potential mediators, is worth further exploration.^127^

**TABLE 3:** Microbial metabolites as biomarkers for cancer diagnosis, therapeutic efficacy assessment and prognostic prediction.

Cancer	Usage	Sample	Method	Sample size	Biomarker	Identification	Test accuracy	Refs.
CRC	Diagnosis	Faecal and serum	Metagenomic sequencing and metabolomic	CRC (*n* = 35) CRA (*n* = 31) HC (*n* = 34)	12 gut microbes	Healthy control vs. CRC	Training cohort: AUC = .919 (.873–.965) Validation cohorts 1: AUC = .864 (.799–.93) Validation cohorts 2: AUC = .817 (.733–.901) Validation cohorts 3: AUC = .773 (.69–.856) Validation cohorts 4: AUC = .869 (.804–.934) Validation cohorts 5: AUC = .746 (.655–.837) Validation cohorts 6: AUC = .761 (.681–.842) Validation cohorts 7: AUC = .727 (.629–.826)	^91^
					Three serum metabolites		Training cohort: AUC = .967 (.932–1) Validation cohorts: AUC = .821 (.761–.882)	
					12 gut microbes and three serum metabolites		Training cohort: AUC = .995 (.986–1) 10‐fold cross validation: AUC = .994 (.984–1)	
						Healthy control vs. CRA	10‐fold cross validation: AUC = .912 (.833–.992)	
		Faecal and serum	Metagenomic sequencing and metabolomic	CRC (*n* = 49) CRA (*n* = 12) HC (*n* = 31)	Eight serum metabolites	Healthy control vs. CRC and CRA	Modelling cohort: AUC = .98 (.94–1) (specificity = .925, sensitivity = .942) Validation cohort: AUC = .92 (specificity = .849, sensitivity = .835)	^119^
						Healthy control vs. CRA	Validation cohort: AUC = .84 (specificity = .849, sensitivity = .632)	
						Healthy control vs. early/mid‐stage (I/ II) CRC	Validation cohort: AUC = .93 (specificity = .849, sensitivity = .882)	
						Healthy control vs. late‐stage (III/IV) CRC	Validation cohort: AUC = .91 (specificity = .849, sensitivity = .842)	
		Faecal	16S rRNA gene sequencing and metabolomic	CRC (*n* = 50) HC (*n* = 50)	Cadaverine	Healthy control vs. CRC	AUC = .764 (.656–.861) (specificity = .8, sensitivity = .7)	^128^
					Putrescine	Healthy control vs. CRC	AUC = .672 (.56–.783) (specificity = .7, sensitivity = .6)	
		Faecal	Metagenomic sequencing and metabolomic	CRC (*n* = 118) CRA (*n* = 140) HC (*n* = 128)	20 gut metabolites	Healthy control vs. CRC	AUC = .8005 (.7457–.8554)	^121^
					11 gut metabolites	Healthy control vs. CRA	AUC = .6853 (.6223–.7482)	
					13 gut metabolites	CRA vs. CRC	AUC = .81 (.7575–.8625)	
					Six gut microbes	Healthy control vs. CRC	AUC = .905 (.8703–.9397)	
					14 gut microbes	Healthy control vs. CRA	AUC = .8408 (.7953–.8864)	
					Six gut microbes	CRA vs. CRC	AUC = .9071 (.8727–.9415)
					Six gut microbes and 11 gut metabolites	Healthy control vs. CRC	AUC = .9417 (.9151–.9683)	
					14 gut microbes and two gut metabolites	Healthy control vs. CRA	AUC = .8759 (.8358–.916)	
					Six gut microbes and four gut metabolites	CRA vs. CRC	AUC = .9375 (.9107–.9642)	
		Faecal	Metagenomic sequencing and metabolomic	LO‐CRC (*n* = 130) LO‐control (*n* = 97) EO‐CRC (*n* = 100) EO‐control (*n* = 114)	32 microbial species	Healthy control vs. LO‐CRC	Testing cohort: AUC = .8453 Validation cohort: AUC = .7817	^122^
					16 gut metabolites		Testing cohort: AUC = .833 Validation cohort: AUC = .7847	
					59 KO genes		Testing cohort: AUC = .8497 Validation cohort: AUC = .8122	
					Three above features integrated		Testing cohort: AUC = .9234 Validation cohort: AUC = .8236	
					49 microbial species	Healthy control vs. EO‐CRC	Testing cohort: AUC = .886 Validation cohort: AUC = .7734	
					36 gut metabolites		Testing cohort: AUC = .8828 Validation cohort: AUC = .7535	
					59 KO genes		Testing cohort: AUC = .8395 Validation cohort: AUC = .7552	
					Three above features integrated		Testing cohort: AUC = .9165 Validation cohort: AUC = .7847	
		Faecal and plasma	Metagenomic sequencing and metabolomic	CRC (*n* = 422) CRA (*n* = 399) HC (*n* = 430)	17 plasma metabolites	Healthy control vs. CRC	Discovery cohort: AUC = .927 (specificity = .936, sensitivity = .802) Validation cohort 1: AUC = .987 (specificity = .884, sensitivity = .991) Validation cohort 2: AUC = .848 (specificity = .813, sensitivity = .785) Validation cohort 3: AUC = .909 (specificity = .888, sensitivity = .845)	^120^
						Healthy control vs. CRA	Discovery cohort: AUC = .968 (specificity = .972, sensitivity = .870) Validation cohort 1: AUC = .955 (specificity = .777, sensitivity = .982) Validation cohort 2: AUC = .838 (specificity = .692, sensitivity = .850) Validation cohort 3: AUC = .725 (specificity = .698, sensitivity = .655)	
	Efficacy			R (*n* = 43) NR (*n* = 29)	19 plasma metabolites	Chemotherapeutic responders vs. non‐responders	AUC = .908 (specificity = .833, sensitivity = .864)	
Biliary tract cancer		Faecal	Metagenomic sequencing and metabolomic	DCB (*n* = 47) NDB (*n* = 41)	Six gut microbes	Anti‐PD‐1/PD‐L1 DCB vs. NDB	Training cohort: AUC = .8969 (.7887–1) Testing cohort: AUC = .7222 (.4586–.9858)	^125^
					Four gut metabolites		Training cohort: AUC = .8625 (.7438–.9812) Testing cohort: AUC = .7531 (.5081–.9981)	
					Three gut microbes and two gut metabolites		Training cohort: AUC = .9594 (.9041–1) Testing cohort: AUC = .8395 (.4586–1)	
Solid cancer tumours	Faecal and plasma	Target metabolomic	R (*n* = 15) NR (*n* = 37)	Propionic acid	Treatment with nivolumab or pembrolizumab R vs. NR	Univariate analyses: HR = .08 (.03–.20) Multivariate analysis: HR = .07 (.03–.19)	^124^
PaCa	Prediction	Serum and plasma	Metabolomic	Diagnosed within 5 years (*n* = 172) control (*n* = 863)	Three microbial metabolites	Diagnosed within 5 years vs. control group	AUC = .64 (.54–.73) adj OR = 1.42 (.94–2.13)	^118^
					Three microbial metabolites and five non‐microbial metabolites		AUC = .79 (.71–.88) adj OR = 3.13 (2.08–4.98)	
					Three microbial metabolites and five non‐microbial metabolites and CA19‐9		Set‐aside test set: AUC = .84 (.76–.91) adj OR = 9.67 (4.56–23.3) Entire set: AUC = .80 (.75–.83) adj OR = 8.44 (5.80–12.20)	

Abbreviations: adj OR, adjusted odds ratio; AUC, area under the curve; CA19‐9, carbohydrate antigen 19‐9; CRA, colorectal adenoma; CRC, colorectal cancer; DCB, durable clinical benefit; EO‐CRC, early‐onset colorectal cancer; HC, healthy control; HR, hazard ratio; KO, Kyoto Encyclopedia of Genes and Genomes (KEGG) orthology; LO‐CRC, late‐onset colorectal cancer; NDB, non‐durable clinical benefit; NR, non‐responders; PaCa, pancreatic cancer; PD‐1, programmed cell death protein 1; PD‐L1, programmed cell death‐ligand 1; R, responders.

In addition to serving as biomarkers, novel intervention strategies based on microbial metabolites offer new perspectives for cancer treatment. Compared to direct utilisation of active microbes (such as pr obiotics and faecal microbiota transplantation), microbial metabolites, that is postbiotics, exhibit several notable advantages, including well‐defined structure, stable dosage, convenience of processing and storage, as well as avoiding the potential issues of biological organisms acquiring antibiotic resistance genes and virulence factors.^129^ By harnessing the bactericidal effects of butyrate on *F. nucleatum*, the administration of sodium butyrate (NaBu) encapsulated in liposomes or prepared as NaBu tablets with an Eudragit S100 coating significantly enhanced the therapeutic efficacy of oxaliplatin in mice with CRC.^130^ Moreover, poly nanoparticles (NPs) loaded with D‐lactate were used to create a nanoformulation that remodelled the immunosuppressive TME by repolarising TAMs and provided a combinatorial strategy for HCC immunotherapy.^42^

Prebiotics and dietary supplements can indirectly influence cancer therapy through modulating the concentration of microbial metabolites. For example, a well‐recognised prebiotic inulin has been found to increase the abundance of SCFAs in mice and boost anti‐PD1 therapy efficacy.^131^ Meanwhile, ginseng polysaccharide, a prebiotic extracted from the traditional Chinese medicine ginseng, promotes the anti‐tumour response to PD‐1 mAb by increasing the level of the microbial metabolite valeric acid and decreasing that of L‐kynurenine, as well as the Kyn/Trp ratio.^132^ However, previous research suggests that inulin can disrupt the intestinal microbiota and promote the progression of HCC through its derivative butyrate.^133^ Another study also indicates that excessive consumption of inulin may lead to an inflammatory state within the body.^134^ Similarly, although a moderate intake of dietary fibre can improve the response to ICB therapy in patients with melanoma,^135^ excessive supplementation may have health risks; for example, a previous study revealed that excessive soluble dietary fibre (20% in feed) caused intestinal flora disorders, accompanied by an increase in faecal butyrate and serum BA, which commonly drive CRC in mice.^136^ These phenomena underscore the importance of the proper use of prebiotics and dietary improvement.

## 4 METHODOLOGICAL FRAMEWORK OF MICROBIAL METABOLOMICS

Currently, microbiome data are mainly obtained using 16S rRNA sequencing and shotgun metagenomic sequencing to study microbial diversity, composition, abundance and functions.^137^ 16S rRNA sequencing is a targeted polymerase chain reaction (PCR) amplification technique focused on the hypervariable regions of bacterial 16S rRNA. PICRUSt and Tax4Fun are 16S‐based tools for predicting bacterial community functions, including Kyoto Encyclopedia of Genes and Genomes (KEGG) pathways and metabolic information. Compared to the former, shotgun metagenomic sequencing allows for microbiota identification down to the species or even strain level. It facilitates in‐depth analyses of gene levels, including gene composition, functions and pathways. HUMAnN is a pipeline designed to profile metabolic pathways abundance and multiple bacteria contributions using metagenomic data. It is important to emphasise that functional and metabolic analyses at the genetic level are merely potential predictions; they cannot reflect actual metabolic activity, and integration with other meta‐omics analyses is required. Metatranscriptomics focuses on the analysis of the collective microbial transcriptome of a community. However, host RNA contamination and RNases present in host‐derived samples pose challenges to procedures of sample collection, storage and preparation during metatranscriptomic research.^138^ Compared to metatranscriptomics, metaproteomics can provide deeper insights into microbial community functions, as not all transcripts can be translated into proteins. However, metaproteomics is an immature field with various shortcomings, including difficulties in protein extraction and data analysis, and incomplete microbial protein databases, making it currently less utilised for microbial analysis.

In addition to microbial profiling, metabolomics, an emerging discipline that has developed rapidly, also contributes greatly to dissecting the functional consequences of the microbiome. Untargeted and targeted metabolomics are recently developed reliable methods for qualitatively, quantitatively and functionally analysing metabolites via a combination of MS and/or NMR technologies. The former allows for a systematic analysis of all metabolites derived from an organism, while the latter focuses on specific metabolites. The identification of metabolites is based on comparing the spectra detected by instruments with those in existing databases. Therefore, the coverage of substances in the database significantly affects the amounts of identifications in metabolomics. Commonly used metabolomics databases include Human Metabolome Database (HMDB), Golm Metabolome Database (GMD), Lipid Maps, MassBank, as well as microbiome‐related metabolic databases such as Human Microbial Metabolome Database (MiMeDB),^139^ Metabolite Origin (MetOrigin),^140^ Microbe‐Mass Spectrometry Search Tool (microbeMASST),^141^ gut Microbe‐Gene (gutMGene)^142^ and a newly developed gut microbiome metabolomics data pipeline.^143^ However, only approximately 10% of metabolite structures can be annotated; it makes comprehensively determining the metabolites that truly impact human health quite challenging.^144^ Recently, reverse metabolomics has been proposed as a novel strategy to broaden the boundaries of metabolites; in this method, tandem MS data are obtained from newly synthesised compounds and searched among public metabolomics data to reveal phenotype associations.^145^ In recent years, artificial intelligence has also been applied to MS data analysis, improving the efficiency and accuracy of metabolomics data analysis to reveal disease‐specific metabolic profiles better.^146^, ^147^

With the rapid development of multi‐omics integrative analysis, striking connections between the microbiome and metabolite profiles collected from the same individuals have gradually been revealed. Correlation analysis, such as Pearson and Spearman analyses, along with multivariate statistical methods like canonical correspondence analysis (CCA), redundancy analysis (RDA), sparse partial least squares (sPLS) and two‐way orthogonal partial least squares (O2PLS) analyses, are the commonly used approaches to statistically investigate intercorrelations and identify salient features in multi‐omics data integration. Furthermore, several data mining algorithms, such as machine learning and deep learning are increasingly being employed in microbial metabolomics studies.^148^, ^149^ Moreover, many microbiome‐related metabolite prediction tools have been developed. For example, the microbe–metabolite interactions‐based metabolic profiles predictor (MMINP) constructed based on the O2‐PLS algorithm, can build models from partial training samples to predict metabolite data from metagenomic data.^150^ In recent years, in addition to the integrative analysis of microbiomes and metabolomics, there has been an increasing number of studies combining metaproteomics or metatranscriptomics.^151^, ^152^, ^153^ These multi‐omics integrative analysis can be utilised for pathway analysis, more precise quantification of microbial metabolite and functional prediction.

However, metabolomics can only reflect the static abundance of metabolites, which is often insufficient to address all the issues in the study of specific metabolic pathways and metabolic networks. The new technology Metabolic Flux, developed based on traditional metabolomics, utilises stable isotopes such as 13C or 15N to label specific metabolite molecules in combination with MS and/or NMR technologies. This allows for the dynamic analysis of intracellular metabolism and the flux distribution of metabolic pathways, helping to resolve issues related to the sources and routes of metabolic products. Currently, stable isotope tracing has become a powerful tool for elucidating microbial metabolism due to its ability to label specific metabolic pathways and molecules.^154^

**FIGURE 5:**
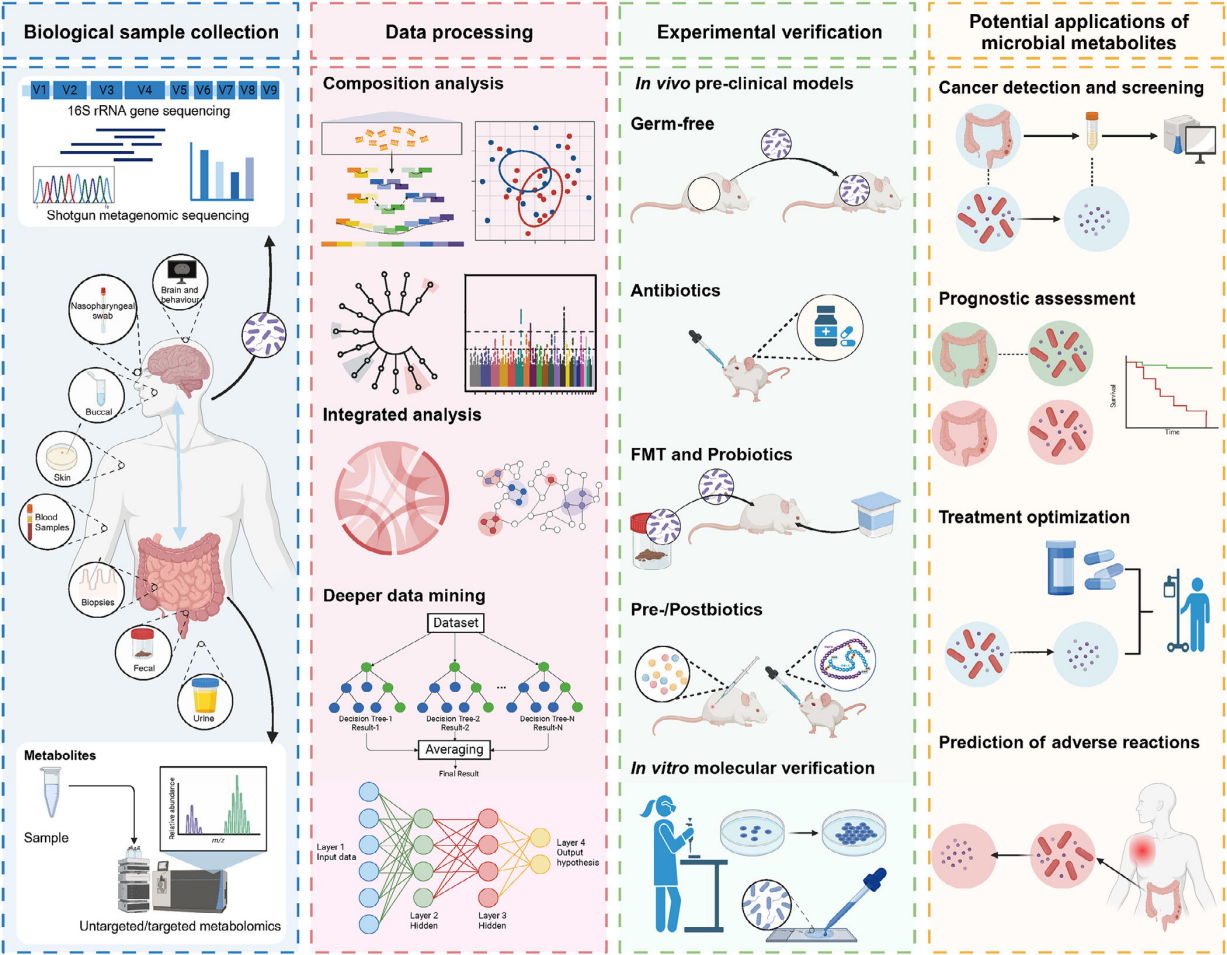
Research method in microbial metabolomics. (A) Biological sample collection. Microbiome information is obtained through the 16S rRNA sequencing and shotgun metagenomic sequencing, while metabolites information is acquired through the use of mass spectrometry (MS) and/or nuclear magnetic resonance (NMR) technologies. (B) Data processing. Through the processes of composition analysis, integrated analysis and deeper data mining, a more thorough analysis and exploration of microbial metabolites can be achieved. (C) Experimental verification. In fundamental studies, many in vivo pre‐clinical models and in vitro molecular verifications are essential for advancing mechanistic understanding. (D) Potential applications of microbial metabolites. Microbial metabolites have the potential to be applied in the diagnosis, prognostic assessment, treatment optimisation and adverse reactions prediction of cancer. FMT, faecal microbiota transplantation.

In conclusion, by conducting correlational and differential analyses of biological data, followed by experimental verification both in vitro and in vivo, we can uncover insights into the functions and mechanisms of microbial metabolites, ultimately aiding in biomarker screening and treatment optimisation, and providing comprehensive insights into microbial metabolomics (Figure 5).

## 5 DISCUSSION

### 5.1 Reasons for the paradoxical functions of microbial metabolites

SCFAs, SBAs, TMAO, inosine, UA, lactic acid and other microbial metabolites have dual effects on tumours, and their concentrations may play a key role in these effects. The contradictory effect of butyrate on tumours is called the ‘butyrate paradox’: a lower concentration of butyrate provides energy to cells through β‐oxidation and promotes cell proliferation and growth, while a higher concentration of butyrate acts as an HDACs inhibitor, thereby inhibiting proliferation and promoting the apoptosis of intestinal epithelial cells.^155^, ^156^ However, previous studies have indicated that relatively high concentrations of butyrate can also promote colorectal and liver cancer development. Therefore, explaining such a paradoxical phenomenon solely based on concentration is difficult. To fully understand the function of microbial metabolites, the circumstances in which they act, such as the type of cancer cells and the TME, cannot be ignored. Despite SBAs are typically seen as promoting colon cancer, they can also induce breast cancer cell apoptosis and inhibit GBC cell proliferation.^33^, ^35^ Moreover, inosine can only induce effective anti‐tumour immunity under sufficient co‐stimulation conditions.^41^ Further investigations are needed to elucidate the diverse mechanisms of microbial metabolites in tumours.

### 5.2 Limitations of microbial metabolite research

Integrating metabolomics and the microbiome enhances our understanding of how microbiota affects the host's metabolism and disease through co‐metabolism. However, several challenges hinder microbial metabolomics studies. Reliable methods are still lacking to accurately identify the primary sources of specific metabolites under physiological conditions during metabolite detection, which may include a particular host organ or a specific type of microorganism in the gut. First, there is a lack of standardised metabolite quenching and extraction methods for microbial metabolomics sample pretreatments.^157^ Second, microbial metabolomics databases are limited to specific microorganisms, primarily yeast and *Escherichia coli*. Thus, establishing more comprehensive standard databases that encompass various microbial metabolomics methods for information integration and dissemination is necessary. Third, reliable methods are still lacking to accurately identify the sources of specific metabolites under physiological conditions during metabolite detection, which may include a particular host organ or a specific type of microorganism in the gut. Finally, the most commonly used 16S rRNA amplicon sequencing and untargeted metabolomics are both relatively quantitative methods; however, relying solely on relative quantification can lead to inaccurate conclusions, making the incorporation of absolute quantification techniques essential for a deeper understanding of host–microbe metabolite interactions.^158^, ^159^

Apart from technical limitations, many blank areas in the field of microbial metabolite research need further research. Current studies primarily focus on the link between specific microbial metabolites and a particular type of cancer, leaving the mechanistic interactions between them unclear. Meanwhile, recent research has also found that probiotics can facilitate a cross‐feeding process with tumour‐enriched bacteria via their metabolites, thereby contributing to tumourigenesis^160^; thus, more extensive and in‐depth studies are needed to elucidate microbial metabolic networks and microbial‐metabolites interactions. Additionally, most research on microbial metabolites has focused on gut metabolites. Since metabolites from intratumoural microbiota may have a more direct impact due to their higher concentrations in the TME, they deserve more attention.^161^, ^162^

### 5.3 Challenges of utilising microbial metabolites in clinical applications

Biomarkers are typically selected through strict matching, aiming to identify accurate and reliable ones by minimising confounding factors. However, in reality, there are significant differences in interindividual health status, diet, the natural environment and the social environment, resulting in unique metabolic changes in the body.^163^ As a result, the chosen biomarkers may face many unavoidable interfering factors in practice. Although metabolomics methods are widely used to study various cancers, most selected biomarkers have not been successfully applied in clinical cancer screening.^164^ Moreover, when selecting biomarkers, people mainly focus on one or a few biomarkers to explain overall changes in the body. However, different diseases or exposures may lead to similar changes in metabolites, making it challenging to use metabolites as biomarkers in clinical settings. For instance, chronic obstructive pulmonary disease (COPD) and lung cancer share similar biomarker patterns. Liver diseases and polyneuropathies show nearly identical biomarker associations.^165^

In addition, there are still many unresolved issues related to the use of microbial metabolites in tumour treatment. Some postbiotics may exhibit paradoxical effects at different concentrations and under various circumstances, leading to several safety concerns regarding their application. Currently, apart from one clinical trial investigating the effects of SCFA supplements on the quality of life and treatment‐related toxicity in subjects receiving abdominopelvic radiotherapy (NCT04700527), the current findings on the therapeutic applications of microbial metabolites are derived primarily from pre‐clinical animal studies. Further studies on human tumours are needed to elucidate the effect of microbial metabolites at certain concentrations and under specific circumstances to explore the therapeutic transition from one‐size‐fits‐all designs to personalised approaches. More in‐depth studies, including toxicology, pharmacodynamics and pharmacokinetics analyses, are essential to determine the safety, benefits and optimal intake of postbiotics for the treatment of cancer.

## 6 CONCLUSION

In conclusion, microbiota‐derived metabolites play important roles in the initiation and progression of tumours. With the advancement of multi‐omics technologies such as microbiome and metabolomics, the revelation of microbial metabolites has led to a leap in understanding tumours from a ‘microbial holistic level’ to ‘precise molecular mechanisms’. Metabolites derived from gut microbiota and intratumoural microbiota influence tumour progression through various mechanisms, including disrupting cellular signalling pathways, triggering oxidative stress, inducing metabolic reprogramming and reshaping the tumour immune microenvironment. Despite numerous unresolved issues in this field, microbial metabolites hold great clinical translational potential for cancer diagnosis, prognosis and treatment. Exploring extensive research across all levels, including fundamental and translational studies and clinical trials, could uncover innovative approaches involving microbial metabolites that could be utilised to advance the development of precision medicine in cancer treatment.

## AUTHOR CONTRIBUTIONS

Na Liu obtained funding and designed the contents. Yu‐Fei Duan, Jia‐Hao Dai, Ying‐Qi Lu and Han Qiao drafted the manuscript. Na Liu performed critical revisions of the manuscript.

## CONFLICT OF INTEREST STATEMENT

The authors declare no conflicts of interest.

## ETHICS STATEMENT

Not applicable.

## CONSENT TO PARTICIPATE

Not applicable.

## CONSENT FOR PUBLICATION

All authors agree to publish.

## CODE AVAILABILITY

Not applicable.

## Data Availability

Not applicable.
